# Synthesis, Crystal Structure, Antibacterial and In Vitro Anticancer Activity of Novel Macroacyclic Schiff Bases and Their Cu (II) Complexes Derived from *S*-Methyl and *S*-Benzyl Dithiocarbazate

**DOI:** 10.3390/molecules28135009

**Published:** 2023-06-26

**Authors:** Mohammed Khaled Bin Break, Tan Yew Fung, May Zie Koh, Wan Yong Ho, Mohamed Ibrahim Mohamed Tahir, Omar Ashraf Elfar, Rahamat Unissa Syed, Weam M. A. Khojali, Turki Mubarak Alluhaibi, Bader Huwaimel, Christophe Wiart, Teng-Jin Khoo

**Affiliations:** 1 Department of Pharmaceutical Chemistry, College of Pharmacy, University of Hail, Hail 55473, Saudi Arabia; we.ali@uoh.edu.sa (W.M.A.K.); s201606288@liveuohedu.onmicrosoft.com (T.M.A.); b.huwaimel@uoh.edu.sa (B.H.); 2 Medical and Diagnostic Research Centre, University of Hail, Hail 55473, Saudi Arabia; ru.syed@uoh.edu.sa; 3 School of Pharmacy, Faculty of Science and Engineering, University of Nottingham Malaysia, Semenyih 43500, Selangor, Malaysiaokamal275@gmail.com (O.A.E.); 4 Division of Biomedical Sciences, School of Pharmacy, Faculty of Science and Engineering, University of Nottingham Malaysia, Semenyih 43500, Selangor, Malaysia; mayzie.koh@gmail.com (M.Z.K.); wanyong.ho@nottingham.edu.my (W.Y.H.); 5 Department of Chemistry, Faculty of Science, University Putra Malaysia, Serdang 43400, Selangor, Malaysia; ibra@upm.edu.my; 6 Department of Pharmaceutics, College of Pharmacy, University of Hail, Hail 55473, Saudi Arabia; 7 Department of Pharmaceutical Chemistry, Faculty of Pharmacy, Omdurman Islamic University, Omdurman 14415, Sudan; 8 Institute for Tropical Biology & Conservation, University Malaysia Sabah, Kota Kinabalu 88400, Sabah, Malaysia; christophewiart@ums.edu.my

**Keywords:** antibacterial, anticancer, copper, crystallography, Schiff base

## Abstract

A series of novel macroacyclic Schiff base ligands and their Cu (II) complexes were synthesised via reacting dicarbonyls of varying chain lengths with *S*-methyl dithiocarbazate (SMDTC) and *S*-benzyl dithiocarbazate (SBDTC) followed by coordination with Cu (II) ions. X-ray crystal structures were obtained for compound **4**, an SBDTC-diacetyl analogue, and **Cu7**, an SMDTC-hexanedione Cu (II) complex. Anticancer evaluation of the compounds showed that **Cu1**, an SMDTC-glyoxal complex, demonstrated the highest cytotoxic activity against MCF-7 and MDA-MB-231 breast cancer cells with IC_50_ values of 1.7 µM and 1.4 µM, respectively. There was no clear pattern observed between the effect of chain length and cytotoxic activity; however, SMDTC-derived analogues were more active than SBDTC-derived analogues against MDA-MB-231 cells. The antibacterial assay showed that *K. rhizophila* was the most susceptible bacteria to the compounds, followed by *S. aureus*. Compound **4** and the SMDTC-derived analogues **3**, **5**, **Cu7** and **Cu9** possessed the highest antibacterial activity. These active analogues were further assessed, whereby **3** possessed the highest antibacterial activity with an MIC of <24.4 µg/mL against *K. rhizophila* and *S. aureus*. Further antibacterial studies showed that at least compounds **4** and **5** were bactericidal. Thus, **Cu1** and **3** were the most promising anticancer and antibacterial agents, respectively.

## 1. Introduction

Schiff bases comprise a group of compounds that are characterised by the presence of an imine functional group. They have been found to possess a wide range of biological activities, including anticancer, antioxidant, antiviral and antibacterial activities [1]. Moreover, the nitrogen atom of the azomethine group in Schiff bases was found to interact with proteins and DNA, which might explain some of the observed biological activities [2]. Schiff bases have also been extensively used in drug design owing to their simple synthesis and solubility in various organic solvents [2]. 

Dithiocarbazates possessing nitrogen–sulfur chelating agents have been heavily studied for their potential bioactivities, such as antibacterial and anticancer activities [3,4,5]. Moreover, their corresponding Schiff base ligands yielded a wide range of biological activities, often resulting from slight modifications to their molecular structures [6,7]. These Schiff base ligands often possess nitrogen–sulfur atoms, which enable them to chelate with a range of metal ions, especially transition metal ions, resulting in metal complexes with enhanced biological activities [8]. Macrocyclic and macroacyclic dithiocarbazate-derived Schiff base ligands were found to potentially exhibit a range of biological applications [9,10]. For instance, a previous study [9] reported the potential anticancer and antibacterial activities of macroacyclic Schiff base ligands derived from *S*-benzyl dithiocarbazate (SBDTC)/*S*-methyl dithiocarbazate (SMDTC) and 2,5-hexanedione along with their corresponding Cu (II) complexes. However, this study was extremely limited and experimented with only two Schiff base ligands, and this made it crucial to further investigate the potential of these promising dithiocarbazate-derived macroacyclic compounds as potential antibacterial/anticancer agents.

Therefore, the main aim of this present study is to synthesise novel macroacyclic Schiff base ligands derived from SBDTC/SMDTC and dialdehyde/diketones. The resulting Schiff base ligands would then be coordinated with Cu (II) ions to produce metal complexes. Dialdehyde/diketones were used to synthesise the Schiff base ligands due to a previous study that demonstrated the high bioactivity of Schiff base ligands derived from SBDTC/SMDTC and 2,5-hexanedione [9]. Moreover, we reasoned that the presence of more than one imine linkage per Schiff base ligand would result in higher biological activities via synergistic effects. It is predicted that having two imine linkages would enhance the binding interactions that are expected to occur between the Schiff base ligand and their respective biological targets, which would lead to a higher binding affinity that would most likely result in more potent biological activity. SBDTC and SMDTC were used to synthesise the Schiff base ligands due to the high number of studies that reported the potent biological activities of SBDTC- and SMDTC-derived Schiff bases ligands [11,12]. Cu (II) ions were considered for complexation due to previous studies which showed that Cu (II) complexes of nitrogen–sulfur ligands are often stable and neutral compounds that possess the ability to easily cross cellular membranes, which is a desirable property for most drugs [13,14]. Furthermore, several studies have reported the high biological activity of Cu (II) complexes, including those derived from dithiocarbazates, and this convinced us further to coordinate our Schiff base ligands with Cu (II) ions [8,15,16]. 

Herein, we describe the synthesis of novel macroacyclic Schiff base ligands and their corresponding Cu (II) complexes as potential anticancer/antibacterial agents. Schiff base ligands were synthesised via a condensation reaction involving SMDTC and SBDTC with 2,3-dialdehyde/diketones of varying chain lengths in order to study the effect of chain length on bioactivity and also discover a potent analogue. The resulting Schiff base ligands were then coordinated with Cu (II) ions. The synthetic compounds were extensively characterised via a variety of techniques, such as IR, NMR, UV-Vis, cyclic voltammetry, elemental analysis and X-ray crystallography, while biological activity was assessed using MTT, disc diffusion, MIC and MBC assays. 

## 2. Results and Discussion

### 2.1. Synthesis, Physical and Spectroscopic Characterisation

**Scheme 1:**
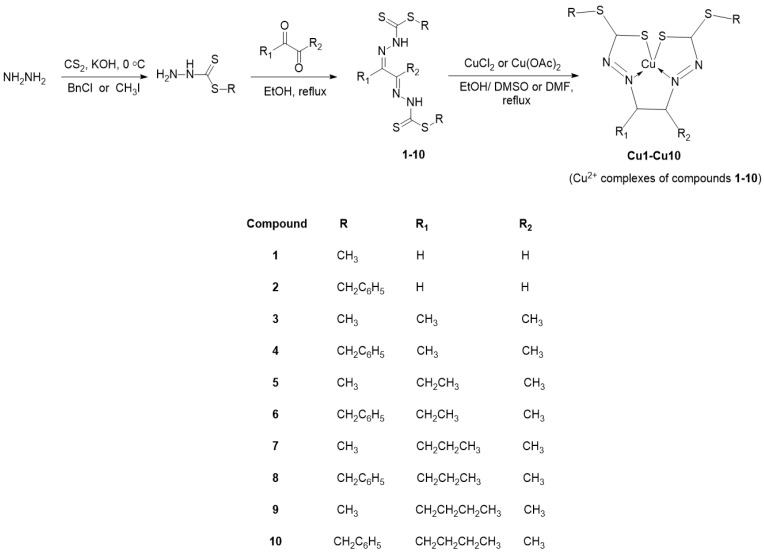
Synthesis of the Schiff base ligands and their corresponding Cu (II) complexes.

Schiff bases and their metal complexes have been known for their interesting biological activities throughout the literature, especially dithiocarbazate-derived Schiff bases and their complexes. Therefore, in this study, a series of dithiocarbazate-derived macroacyclic Schiff base ligands along with their Cu (II) complexes were synthesised, and their potential as effective anticancer and antimicrobial agents was investigated. The synthesis methodology is shown in Scheme 1.

Synthesis of the Schiff base ligands involved reacting specific dicarbonyl compounds with SMDTC or SBDTC in absolute ethanol. The metal complexes involved reacting the ligands with a Cu (II) salt solution in a 1:1 ratio. The reactions were straightforward, and the ligands and complexes were obtained directly without further purification. The synthetic compounds were later subjected to physical, spectral, electrochemical and crystallographic analyses.

NMR spectral analysis (See Supplementary Material) of the compounds showed that the Schiff base ligands were obtained as intended. In general, the NMR spectra showed peaks for both the dithiocarbazate and diketone moieties, while the peak integrals confirmed that the ratio between the dithiocarbazate and diketone moieties in the formed Schiff base ligands was 2:1. SMDTC-derived Schiff base ligands showed characteristic singlet peaks at around 2.5 ppm corresponding to the methyl protons of the dithiocarbazate moiety. On the other hand, the SBDTC-derived Schiff base ligands showed characteristic singlet peaks at around 4.5 ppm and multiplet peaks at around 7.4 ppm corresponding to the methylene protons and aromatic protons of the dithiocarbazate moiety, respectively. Moreover, the NMR spectra of all ligands showed broad singlet peaks at around 12.8 ppm corresponding to the amino proton of the dithiocarbazate moiety. Glyoxal-derived Schiff base ligands showed a characteristic singlet peak at around 7.8 ppm corresponding to the proton adjacent to the imine group, while butanedione-derived Schiff base ligands showed a singlet peak at around 2.2 ppm corresponding to the methyl groups of the diketone moiety. Pentanedione-, hexanedione- and heptanedione-derived Schiff base ligands also showed characteristic triplet and multiplet peaks corresponding to the methylene groups of the diketone moieties. Therefore, NMR spectral analysis confirmed the formation of the intended Schiff base ligands. 

FTIR spectral analysis (See Supplementary Material) was also used in order to characterise the synthetic Schiff base ligands and their corresponding metal complexes. The IR spectra of the Schiff bases showed peaks at around 1500–1600 cm^−1^ corresponding to the imine (C=N) functional groups, which proved the formation of the ligands. The metal complexes’ IR spectra also showed imine group peaks at around 1500–1600 cm^−1^; however, a slight red shift was observed in the metal complexes’ peaks relative to that of the corresponding ligands, which indicated coordination of the imine groups with Cu (II) ions. Furthermore, the metal complexes’ IR spectra showed (N–N) peaks at higher absorption frequencies than those belonging to the corresponding Schiff base ligands due to a decrease in the repulsion that exists between the nitrogen atoms. It is suggested that this decrease in repulsion between the nitrogen atoms is due to the coordinate bond that forms between the imine group and Cu (II) ion; thus, the imine group electrons are no longer in close proximity to the adjacent nitrogen atom’s electrons, resulting in less repulsion [6]. Therefore, these shifts in the absorption frequencies of (C=N) and (N–N) after complexation proved the formation of the intended metal complexes in addition to further confirming the involvement of the imine group in coordination with the metal ion. Elemental analysis of the metal complexes corroborated the data obtained from FTIR spectral analysis and further proved the formation of the metal complexes in a metal:ligand ratio of 1:1; however, it is crucial to note that in some metal complexes, the elemental analysis data deviated largely from the calculated data and were thus excluded. These deviations between the calculated and found elemental analysis data might indicate the formation of polymeric chains or multinuclear copper complexes linked together via salt bridges, and such structures result in unpredictable elemental analysis data that are slightly challenging to interpret. 

UV-Vis spectral analysis of the Schiff base ligands and complexes showed absorption bands at around 280 nm, probably resulting from the ligands’ aromatic electron transitions (*π*→*π^∗^*) and non-bonding electrons (n→*π^∗^*) present in the imine group of the complexes. Moreover, the bands at around 350 nm for the complexes may have been due to electron transitions from the donor atoms’ pπ orbitals to the metal ion’s d-orbitals. Finally, the unique bands at around 500 nm for the metal complexes were attributed to d_x_^2^_−y_^2^→d_xz−yz_ and are indicative of a square pyramidal structure, or the bands may be assigned to the ^4^T_1g_(F)→^4^A_2g_(F) d–d transition, indicating a metal complex with a distorted tetrahedral geometry [17,18,19]. It is also crucial to note that the absence of absorption bands at higher than 600 nm for metal complexes suggests the presence of large crystal-field splitting [20]. 

Therefore, in general, NMR, FTIR, elemental analysis, melting point measurement and UV-Vis spectroscopy confirmed the successful formation of the intended Schiff base ligands and their respective Cu (II) complexes in a metal:ligand ratio of 1:1. Analysis via X-ray crystallography was conducted to further confirm the synthetic compounds’ structures. 

### 2.2. X-ray Crystal Structure Description of Compound ***4*** and Metal Complex ***Cu7***

Single crystals were grown for ligand **4** and metal complex **Cu7** via the slow evaporation method. Their key crystallographic parameters are summarised in Table 1, while important bond lengths and angles for both compounds are summarised in Table 2.

#### 2.2.1. Compound **4** Crystal Structure 

**Figure 1:**
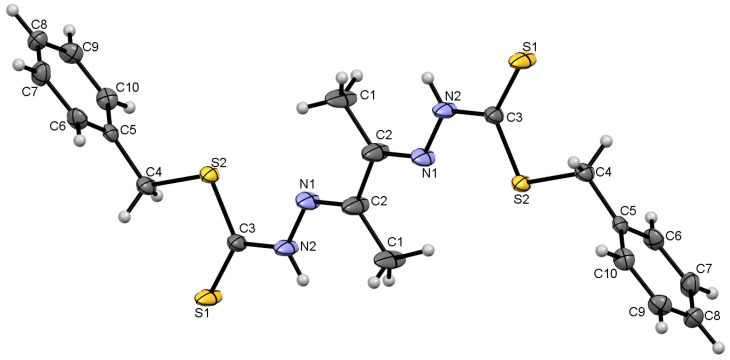
ORTEP diagram of **4** (at 50% probability displacement ellipsoids) with atomic numbering scheme.

An ORTEP diagram of compound **4** with an atomic numbering scheme is shown in Figure 1, while important bond lengths and angles are summarised in Table 2. The ligand crystallised in the monoclinic crystal system with the *P*2_1_/*n* space group. The molecule was found to be mostly planar, except for the benzene rings, which were twisted and almost perpendicular to the rest of the molecule with torsion angles of 87.3° (C6–C10–C4–S2). The (C3–S2) single bond had a length of 1.735 Å, while (C3–S1) had a shorter bond length of 1.659 Å, which indicates the presence of a double bond and that the ligand exists in its thione tautomer. Moreover, (C2–N1) had a bond length of 1.294 Å and was shorter than (C3–N2), which had a bond length of 1.352 Å. This indicates the presence of a double bond in (C2–N1) and that it belongs to the imine group, which is characteristic of Schiff base compounds. 

**Figure 2:**
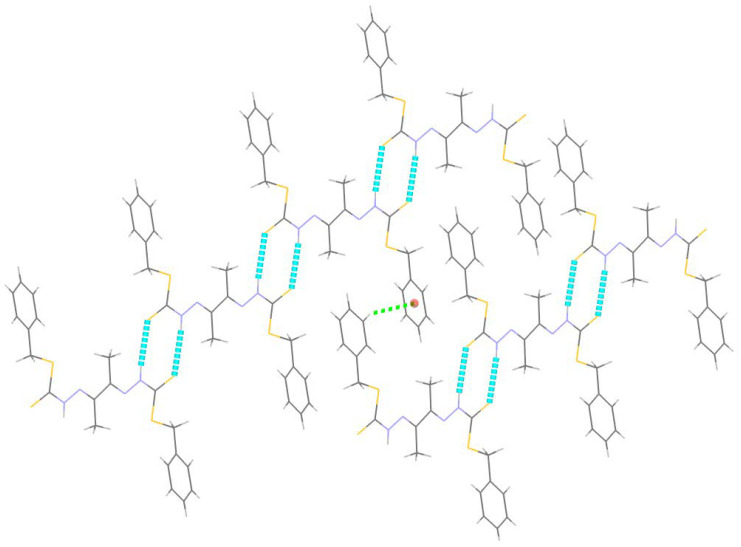
Two-dimensional hydrogen bond network of compound **4** molecules. Blue dotted lines represent S···H hydrogen bonds while the green dotted line represents a C–H···π interaction.

Analysis of interactions between molecules of the crystal structure revealed the presence of hydrogen bond and C–H···π interactions, and details of these interactions are summarised in Table 3. Pairs of N2–H2···S1 hydrogen bonds linked the molecules, resulting in the formation of *R*_2_^2^ (8) ring motifs. The N2–H2···S1 interactions along with the C–H···π interactions resulted in hydrogen bond linkages in the molecular packing (Figure 2). It should be noted that S···H hydrogen bonds are extremely weak, so it seems that further ordering of the crystal lattice was provided by interactions between different layers of the molecular packing. 

#### 2.2.2. Compound **Cu7** Crystal Structure 

**Figure 3:**
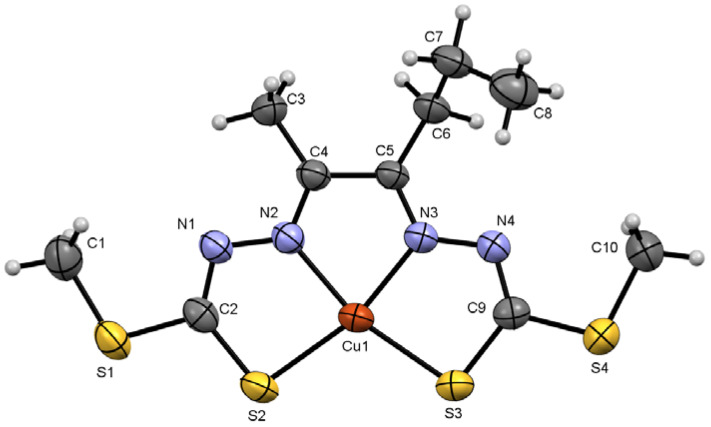
ORTEP diagram of **Cu7** (at 50% probability displacement ellipsoids) with atomic numbering scheme.

An ORTEP diagram of **Cu7** with the atomic numbering scheme is shown in Figure 3, while important bond lengths and angles are summarised in Table 2. The metal complex crystallised in the orthorhombic crystal system with the *Pbca* space group. The Cu (II) ion was found to be bonded to two nitrogen atoms (Cu1–N2 = 1.962 Å; Cu1–N3 = 1.963 Å) and two sulfur atoms (Cu1–S2 = 2.247 Å; Cu1–S3 = 2.243 Å). The structure was essentially planar except for an ethyl group that was almost perpendicular to the rest of the molecule with a torsion angle of 83.1° (C4–C5–C6–C7). The coordination geometry of the metal complex was nearly square planar but with significant tetrahedral distortion, and this was evident from the coordinate angles of the *trans* pairs of donor atoms (N–Cu–S), which were found to be 164.4° (N3–Cu1–S2) and 165° (N2–Cu1–S3) rather than the ideal 180° angles of square planar coordination geometry. The coordination bonds between Cu (II) and the Schiff base ligand resulted in the formation of three nearly planar fused rings around the metal ion: (Cu1–S2–C2–N1–N2), (Cu1–S3–C9–N4–N3) and (Cu1–N2–C4–C5–N3). 

The (C9–S3) coordination bond was found to possess a bond length of 1.752 Å, which is similar to that of the single bond (C9–S4), which has a length of 1.736 Å; this indicates that the ligand coordinates with the Cu (II) ion via its thiol tautomer. Moreover, the bond length of (C9–N4) was found to be 1.303 Å, which is similar to that of the imine group (C5–N3), which possesses a bond length of 1.292 Å, and that further indicates the presence of the ligand in its thiol tautomer upon coordination with Cu (II). 

**Figure 4:**
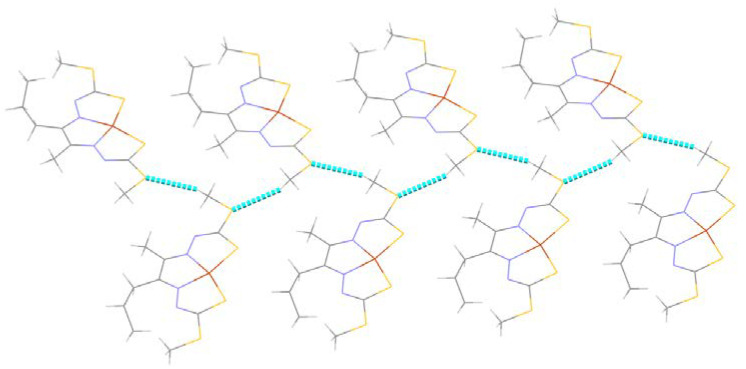
Two-dimensional hydrogen bond network of **Cu7** molecules. Blue dotted lines represent S···H hydrogen bonds.

Analysis of interactions between molecules of the complex showed the presence of hydrogen bond interactions, and details of such interactions are summarised in Table 4. Molecules of the crystal were linked via C1–H1C···S1 hydrogen bond links, resulting in a 2-dimensional supramolecular network (Figure 4). 

### 2.3. Cyclic Voltammetry Analysis of the Metal Complexes

**Figure 5:**
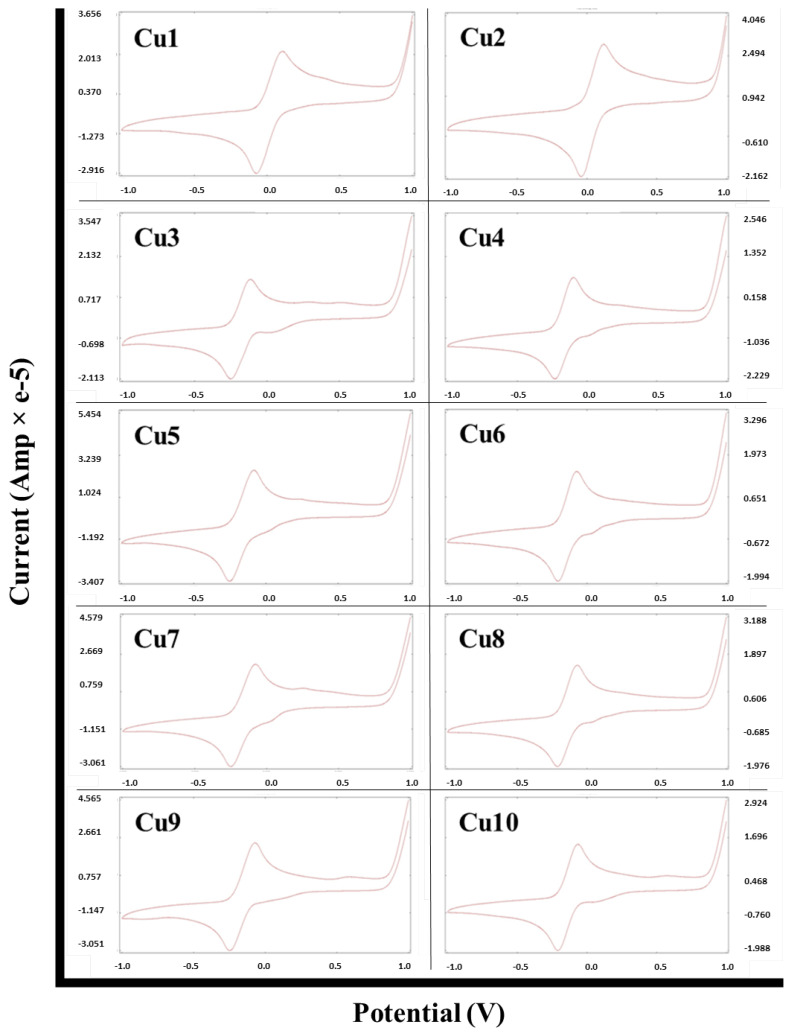
Cyclic voltammograms of Cu (II) complexes at 28 mM in anhydrous oxygenated DMF containing 0.1 M tetrabutylammonium perchlorate as the supporting electrolyte. Working electrode: glassy carbon; counter electrode: Pt wire; reference electrode: Ag/AgCl, scan rate: 100 mV/s.

The redox properties of a certain compound may be utilised to assess its electron-transfer potential. Therefore, the electrochemical behaviours of the Cu (II) Schiff base complexes were studied via cyclic voltammetry and were found to exhibit very similar cyclic voltammograms (Figure 5). The resulting potentials are summarised in Table 5. It can be observed that metal complexes showed a cathodic one-electron reduction peak (*E*_pc_) in the range of −0.261 V to −0.043 V, and these peaks could be attributed to the Cu (II)/Cu (I) reduction process. The metal complexes’ voltammograms also showed an anodic one-electron oxidation peak (*E*_pa_) in the range of −0.115 V to 0.120 V, and these peaks could be attributed to the Cu (I)/Cu (II) oxidation process. The Δ*E*_p_ values (>0.059 V) indicated that the redox processes for the metal complexes are quasi-reversible [21].

### 2.4. Biological Studies

#### 2.4.1. In Vitro Anticancer Studies

The synthetic ligands and their corresponding Cu (II) complexes were screened against 2 breast cancer cell lines, namely, MCF-7 and MDA-MB-231, in order to assess their cytotoxic activity. Results of the MTT cell viability assay are summarised in Table 6. The glyoxal-derived Schiff base ligands, **1** and **2**, were the most active ligands against MCF-7 cells, with IC_50_ values of 6.9 µM and 2.6 µM, respectively. Increasing the chain length by 2 carbon atoms resulted in a decrease in activity, as can be seen for compounds **3** and **4**. However, further increases in chain length resulted in a gradual increase in activity, and that was clearly evident from the decreasing IC_50_ values of compounds **5**–**10**. There was generally no significant difference in activity against MCF-7 cells between SMDTC- and SBDTC-derived ligands, and the only exception to that trend was the difference in activity observed between **3** and **4**. Coordination of the ligands to Cu (II) ions resulted in significantly enhanced cytotoxic activity for ligands **1** and **4**, whereby **Cu1** was found to possess the highest activity against MCF-7 cells with an IC_50_ value of 1.7 µM. This enhanced activity may be explained via the Overtone concept and chelation theory, which states that upon complexation, there is an increase in the delocalisation of π-electrons over the chelate ring, resulting in a decrease in the polarity of the Cu (II) ion; in addition, the metal ion’s positive charge is shared with the donor groups of the chelate ring, which increases the lipophilicity of the metal ion, resulting in enhanced lipophilicity. This enhanced lipophilicity results in better penetration of the complexes into the cell membranes. The complexes may have also exerted their bioactivity via disrupting the cells’ respiration and protein synthesis processes [22]. **Cu5** and **Cu8** showed similar cytotoxic activity to their ligands; however, the activity of the other ligands was found to decrease after complexation with Cu (II) ions. This decrease in activity may have been due to the low solubility of the resulting metal complexes. 

In the case of MDA-MB-231 cells, the synthetic ligands did not show any specific trend or pattern with regards to their activity against the cancer cells. However, there seemed to be a significant difference between the cytotoxic activity of SMDTC- and SBDTC-derived ligands. SMDTC-derived ligands were mostly found to possess higher activity than their corresponding SBDTC-derived ligands, and this could be due to steric hindrance by the large benzene ring of the SBDTC moiety, resulting in inadequate binding with the target receptor. Coordination of the ligands to Cu (II) ions resulted in the most active compound, **Cu1**, which possessed an IC_50_ value of 1.4 µM. Moreover, **Cu6** demonstrated much higher activity than its ligand, while **Cu4** was slightly more active than its ligand. The reason behind this enhanced activity might be explained via the Overtone concept and chelation theory in addition to the complexes’ ability to disrupt cellular processes, as has been mentioned earlier [22]. **Cu5** and **Cu8** had similar activity to their ligands, and this phenomenon was also observed against MCF-7 cells. However, the remaining complexes possessed lower activity than their corresponding ligands, which may have been due to the complexes’ poor solubility. 

Therefore, it can be deduced that **Cu1** is the most active compound across both cell lines and seems to be a highly promising cytotoxic agent. There also seems to be no clear correlation between chain length and cytotoxic activity, but SMDTC-derived ligands seemed to possess higher activity against MDA-MB-231 cells. Moreover, it is crucial to note that most of the synthetic compounds exhibited higher cytotoxic activity than that of cisplatin (positive control), which further reflects the high potential of these synthetic compounds. 

#### 2.4.2. Antibacterial Studies 

The synthetic Schiff base ligands and their metal complexes were investigated for their antibacterial activity. The compounds were first screened against Gram-positive bacteria (*S. aureus*, *B. cereus* and *K. rhizophila*) and Gram-negative bacteria (*E. coli*, *P. aeruginosa* and *C. freundii*) using a qualitative disc diffusion assay. Compounds that demonstrated significant activity in the disc diffusion assay were further assessed quantitatively by measuring their minimum inhibitory concentration (MIC) and minimum bactericidal concentration (MBC) via the broth microdilution method.

##### Qualitative Antibacterial Studies 

Results of the antibacterial disc-diffusion assay are summarised in Table 7. Overall, only a few Schiff base ligands and complexes were found to be active against the bacteria under investigation. In the case of Gram-positive bacteria, there were some compounds that showed activity against *S. aureus* and *K. rhizophila*, but *K. rhizophila* was the one that was specifically inhibited the most. Compounds **3** and **5** showed the highest antibacterial activity and inhibited *K. rhizophila* with inhibition zones of 25 mm and 22 mm, respectively. The activity of **3** and **5** against *K. rhizophila* was even greater than that of chloramphenicol (positive control), which had a smaller inhibition zone of 19.3 mm. There was no significant activity observed against *B. cereus*. 

Gram-negative bacteria were not significantly affected by the Schiff base ligands and complexes. This may have been due to the structural features of Gram-negative bacteria, which consist of an extra outer layer on top of the peptidoglycan that might have been impermeable to the synthetic compounds.

It is crucial to note that most of the active compounds were derived from SMDTC rather than SBDTC, and this could be due to steric hindrance by the larger benzene ring in SBDTC-derived compounds, resulting in weaker binding with the target, which led to lower bioactivity. On the other hand, the methyl group in SMDTC-derived compounds is smaller and did not seem to hinder any potential interactions with the target. There was also a loss in activity after complexation for the active compounds **3**, **4** and **5**, and this could be due to the lower solubility of the resulting Cu (II) complexes. Moreover, there seemed to be no certain pattern or trend between the compounds’ chain length and antibacterial activity.

##### Quantitative Antibacterial Assay 

Compounds that showed activity in the disc diffusion assay were further assessed to investigate their MIC and MBC values. The MIC values for the active compounds against *S. aureus* and *K. rhizophila* are summarized in Table 8. Compound **3** was found to be the most active compound with an MIC value of <24.4 µg/mL against *S. aureus* and *K. rhizophila*, and this was followed by metal complexes **Cu7** and **Cu9** that also exhibited an MIC value of <24.4 µg/mL, but against *K. rhizophila* only. It is interesting to note that these highly active compounds had MIC values that were comparable to that of chloramphenicol, which further highlights their strong antibacterial activity. Moreover, the MIC values further confirmed that the active compounds specifically targeted *K. rhizophila*. 

On the basis of the MBC/MIC ratio, it can be deduced whether a certain active compound is bactericidal (MBC/MIC ≤ 4) or bacteriostatic (MBC/MIC > 4) [23]. In this current study, it was found that the MBC values of **4** and **5** were higher by twofold than the corresponding MIC values against *K. rhizophila*, while the MBC/MIC ratio for the other active compounds will require further testing to determine their MIC more accurately. Therefore, these results suggest that at least compounds **4** and **5** are bactericidal. 

## 3. Materials and Methods

### 3.1. Chemistry

#### 3.1.1. General 

Reagents of analytical grade were used as received from their commercial sources. Benzyl chloride was obtained from ACROS Organics (Morris Plains, NJ, USA), potassium hydroxide from Riendemann Schmidt (Kajang, Malaysia), carbon disulfide from Fisher Scientific (Chino, CA, USA) and copper (II) acetate from R&M Chemicals (Essex, UK). Hydrazine hydrate, iodomethane, glyoxal, 2,3-hexanedione, 2,3-heptanedione, 2,3-pentanedione, 2,3-butanedione and copper (II) chloride were obtained from Merck (Darmstadt, Germany). Absolute ethanol, 95% ethanol, dimethyl sulfoxide, acetone and dimethylformamide were obtained from RCI Labscan (Bangkok, Thailand). Elemental analysis was conducted via a Vario MACRO CUBE CHNS analyser (Germany), while IR spectra were recorded using a PerkinElmer FTIR spectrophotometer (Waltham, MA, USA). Melting points were measured using an Electrothermal IA9100 melting point apparatus (Essex, UK). 

^1^H NMR spectra were obtained via a Bruker FT-NMR 300 MHz (Germany). TLC was performed via 60F aluminium silica gel aluminium plates with a 254 nm fluorescent indicator from Merck (Darmstadt, Germany). UV-Vis spectroscopy was performed via a Biochrom UltroSpec 8000 spectrophotometer (Cambridge, UK).

#### 3.1.2. Synthesis 

##### General Method for Schiff Base Ligands Synthesis 

SMDTC and SBDTC were synthesised as previously reported [17]. SMDTC or SBDTC (0.02 mol) was dissolved in 20–40 mL of absolute ethanol by heating at around 50 °C followed by mixing it with a solution of diketone/dialdehyde (0.01 mol) in 10 mL absolute ethanol. The resulting mixture was heated and stirred gently for 10 to 15 min. To ensure complete reaction, the mixture was left to stir overnight but without heating. The precipitate was then filtered off and washed several times with absolute alcohol and recrystallized whenever possible. The resulting Schiff base was finally dried in a desiccator for 24 h. 

The dicarbonyl compounds used were glyoxal, 2,3-butanedione, 2,3-pentanedione, 2,3-hexanedione and 2,3-heptanedione.

##### General Method for Schiff Base Cu (II) Complexes Synthesis

Schiff-base ligands (0.005 mol) were prepared by dissolving them in 20 mL of absolute ethanol, dimethylsulfoxide (DMSO) or dimethylformamide (DMF). To this solution, Cu(OAc)_2_ or CuCl_2_ (0.005 mol) dissolved in 20 mL of absolute ethanol was added, and the resulting mixture was heated at around 50 °C and left to stir. The resulting precipitate was obtained via vacuum filtration, washed with ethanol and dried in a desiccator.

*SMDTC-glyoxal* (**1**)

Yield: 85%; mp: 198 °C; IR (KBr, cm^−1^): 3110 (N–H), 2959, 1502 (C=N), 1266, 1105, 1039 (C=S), 964, 691 (N–N); ^1^H NMR (300 MHz, DMSO-*d_6_*, *δ*(ppm)): 13.575 (s, 2H, 2 × –N–N**H**–CS–), 7.923 (s, 2H, 2 × –N=C**H–**), 2.568 (s, 6H, 2 × –C**H_3_**); UV-Vis (λ_max_, nm) in DMSO: 257, 349.

*Cu–SMDTC–glyoxal* (**Cu1**)

Yield: 80%; mp: >300 °C; IR (KBr, cm^−1^): 2919, 1500 (C=N), 1407, 1307, 958, 940, 736 (N–N); analytical calculated for Cu(C_6_H_8_N_4_S_4_)(H_2_O): %C (20.83), %H (2.91), %N (16.19), %S (37.07); found: %C (21.49), %H (2.31) %N (15.75), %S (35.41); UV-Vis (λ_max_, nm) in DMSO: 274, 344, 526.

*SBDTC–glyoxal* (**2**)

Yield: 76%; mp: 188 °C; IR (KBr, cm^−1^): 3118 (N–H), 1601 (C=N), 1496, 1401, 1270, 1103, 1040 (C=S), 914, 718 (N–N); ^1^H NMR (300 MHz, DMSO-*d_6_*, *δ*(ppm)): 13.549 (s, 2H, 2 × –N–N**H**–CS–), 7.818 (s, 2H, 2 × –N=C**H–**), 7.408–7.300 (m, 10H, aromatic protons), 4.470 (s, 4H, 2 × S–C**H_2_**–); UV-Vis (λ_max_, nm) in DMSO: 255, 361.

*Cu–SBDTC–glyoxal* (**Cu2**)

Yield: 90%; mp: 225 °C; IR (KBr, cm^−1^): 1600 (C=N), 1402, 1327, 1138, 991, 950, 762 (N–N); analytical calculated for Cu(C_18_H_16_N_4_S_4_)(H_2_O): %C (43.40), %H (3.64), %N (11.25), %S (25.74); found: %C (43.21), %H (3.14) %N (11.05), %S (25.26); UV-Vis (λ_max_, nm) in DMSO: 267, 334, 532.

*SMDTC–Butanedione* (**3**)

Yield: 37%; mp: 204 °C; IR (KBr, cm^−1^): 3183 (N–H), 2920, 1686 (C=N), 1477, 1291, 1140, 1068 (C=S), 958, 704 (N–N); ^1^H NMR (300 MHz, DMSO-*d_6_*, *δ*(ppm)): 12.556 (s, 2H, 2 × –N–N**H**–CS–), 2.511 (s, 6H, 2 × S–C**H_3_**), 2.241 (s, 6H, 2 × –C**H_3_**); UV-Vis (λ_max_, nm) in DMSO: 278, 346.

*Cu–SMDTC–Butanedione* (**Cu3**)

Yield: 61%; mp: 220 °C; IR (KBr, cm^−1^): 1635 (C=N), 1390, 1292, 1030, 957, 928, 790 (N–N); analytical calculated for Cu(C_8_H_12_N_4_S_4_): %C (26.99), %H (3.40), %N (15.74), %S (36.02); found: %C (24.94), %H (2.89) %N (16.52), %S (37.47); UV-Vis (λ_max_, nm) in DMSO: 283, 326, 513.

*SBDTC–Butanedione* (**4**)

Yield: 87%; mp: 200 °C; IR (KBr, cm^−1^): 3179 (N–H), 2920, 1687 (C=N), 1472, 1296, 1137, 1060 (C=S), 946, 778 (N–N); ^1^H NMR (300 MHz, DMSO-*d_6_*, *δ*(ppm)): 12.600 (s, 2H, 2 × –N–N**H**–CS–), 7.419–7.240 (m, 10H, aromatic protons), 4.457 (s, 2H, 2 × S–C**H_2_**–), 2.186 (s, 6H, 2 × –C**H_3_**); UV-Vis (λ_max_, nm) in DMSO: 280, 351.

*Cu–SBDTC–Butanedione* (**Cu4**)

Yield: 50%; mp: 200 °C; IR (KBr, cm^−1^): 2919, 1637 (C=N), 1385, 1030, 921, 697 (N–N); UV-Vis (λ_max_, nm) in DMSO: 271, 330, 506.

*SMDTC–Pentadione* (**5**)

Yield: 70%; mp: 176 °C; IR (KBr, cm^−1^): 3178 (N–H), 2916, 1621 (C=N), 1479, 1290, 1146, 1056 (C=S), 960, 811 (N–N); ^1^H NMR (300 MHz, DMSO-*d_6_*, *δ*(ppm)): 12.717 (s, 1H, –N–N**H**–CS–), 12.540 (s, 1H, –N–N**H**–CS–), 2.858 (q, 2H, C**H_2_**–CN–, *J* = 7.5 Hz), 2.506 (s, 6H, 2 × S–C**H_3_**), 2.229 (s, 3H, –CN–C**H_3_**), 1.02 (t, 3H, C**H_3_**CH_2_–, *J* = 7.2 Hz); UV-Vis (λ_max_, nm) in DMSO: 263, 343.

*Cu–SMDTC–Pentadione* (**Cu5**)

Yield: 80%; mp: 200 °C; IR (KBr, cm^−1^): 2921, 1618 (C=N), 1420, 1297, 1110, 960, 826 (N–N); UV-Vis (λ_max_, nm) in DMSO: 260, 329, 507.

*SBDTC–Pentadione* (**6**)

Yield: 80%; mp: 180 °C; IR (KBr, cm^−1^): 3153 (N–H), 1602 (C=N), 1478, 1294, 1142, 1052 (C=S), 980, 779 (N–N); ^1^H NMR (300 MHz, DMSO-*d_6_*, *δ*(ppm)): 12.785 (s, 1H, –N–N**H**–CS–), 12.594 (s, 1H, –N–N**H**–CS–), 7.426–7.311 (m, 10H, aromatic protons), 4.508 (s, 2H, S–C**H_2_**–), 4.386 (s, 2H, S–C**H_2_**–), 2.805 (q, 2H, C**H_2_**–CN–, *J* = 3.0 Hz), 2.173 (s, 3H, –CN–C**H_3_**), 0.970 (t, 3H, C**H_3_**CH_2_–, *J* = 7.2 Hz); UV-Vis (λ_max_, nm) in DMSO: 262, 343.

*Cu–SBDTC–Pentadione* (**Cu6**)

Yield: 90%; mp: >300 °C; IR (KBr, cm^−1^): 1600 (C=N), 1402, 1265, 1135, 990, 965, 790 (N–N); analytical calculated for Cu(C_21_H_22_N_4_S_4_)(H_2_O)_2_: %C (45.18), %H (4.69), %N (10.04), %S (22.97); found: %C (45.44), %H (4.11), %N (10.76), %S (22.54); UV-Vis (λ_max_, nm) in DMSO: 272, 324, 510.

*SMDTC–Hexadione* (**7**)

Yield: 74%; mp: 178 °C; IR (KBr, cm^−1^): 3193 (N–H), 1594 (C=N), 1479, 1291, 1148, 1066 (C=S), 960, 894 (N–N); ^1^H NMR (300 MHz, DMSO-*d_6_*, *δ*(ppm)): 12.769 (s, 1H, –N–N**H**–CS–), 12.533 (s, 1H, –N–N**H**–CS–), 2.829 (t, 2H, C**H_2_**–CN–, *J* = 7.8 Hz), 2.512 (s, 3H, S–C**H_3_**), 2.502 (s, 3H, S–C**H_3_**), 2.228 (s, 3H, –CN–C**H_3_**), 1.474 (m, 2H, CH_3_C**H_2_**CH_2_–), 0.926 (t, 3H, C**H_3_**CH_2_–, *J* = 7.2 Hz); UV-Vis (λ_max_, nm) in DMSO: 271, 345.

*Cu–SMDTC–Hexadione* (**Cu7**)

Yield: 79%; mp: 208 °C; IR (KBr, cm^−1^): 2962, 1523 (C=N), 1304, 1033, 994, 927 (N–N); UV-Vis (λ_max_, nm) in DMSO: 270, 326, 506.

*SBDTC–Hexadione* (**8**)

Yield: 80%; mp: 164 °C; IR (KBr, cm^−1^): 3166 (N–H), 1602 (C=N), 1473, 1291, 1146, 1060 (C=S), 958, 778 (N–N); ^1^H NMR (400 MHz, DMSO-*d_6_*, *δ*(ppm)): 12.832 (s, 1H, –N–N**H**–CS–), 12.618 (s, 1H, –N–N**H**–CS–), 7.410–7.250 (m, 10H, aromatic protons), 4.458 (s, 2H, S–C**H_2_**–), 4.444 (s, 2H, S–C**H_2_**–), 2.777 (t, 2H, C**H_2_**–CN–, *J* = 7.6 Hz), 2.500 (t, 2H, C**H_2_**–CN, *J* = 1.6 Hz), 2.170 (s, 3H, –CN–C**H_3_**), 1.418 (m, 2H, CH_3_C**H_2_**CH_2_–), 0.834 (t, 3H, C**H_3_**CH_2_–, *J* = 7.5 Hz); UV-Vis (λ_max_, nm) in DMSO: 269, 350.

*Cu–SBDTC–Hexadione* (**Cu8**)

Yield: 93%; mp: >300 °C; IR (KBr, cm^−1^): 1600 (C=N), 1402, 1050, 1024, 994, 789 (N–N); UV-Vis (λ_max_, nm) in DMSO: 272, 324, 509.

*SMDTC–Heptadione* (**9**)

Yield: 93%; mp: >300 °C; IR (KBr, cm^−1^): 3189 (N–H), 2927, 1594 (C=N), 1479, 1133, 1070 (C=S), 961, 820 (N–N); ^1^H NMR (400 MHz, DMSO-*d_6_*, *δ*(ppm)): 12.702 (s, 2H, –N–N**H**–CS–), 2.908 (t, 2H, C**H_2_**–CN–, *J* = 8.0 Hz), 2.561 (s, 3H, S–C**H_3_**), 2.553 (s, 3H, S–C**H_3_**), 2.282 (s, 3H, –CN–C**H_3_**), 1.479–1.372 (m, 4H, CH_3_C**H_2_**C**H_2_**–), 0.937 (t, 3H, C**H_3_**CH_2_–, *J* = 7.2 Hz); UV-Vis (λ_max_, nm) in DMSO: 272, 360.

*Cu–SMDTC–Heptadione* (**Cu9**)

Yield: 89%; mp: >300 °C; IR (KBr, cm^−1^): 2956, 1601 (C=N), 1426, 1100, 1032, 957, 925, 848 (N–N); UV-Vis (λ_max_, nm) in DMSO: 262, 326, 502.

*SBDTC–Heptadione* (**10**)

Yield: 83%; mp: 172 °C; IR (KBr, cm^−1^): 3153 (N–H), 1602 (C=N), 1473, 1401, 1148, 1060 (C=S), 777 (N–N); ^1^H NMR (400 MHz, DMSO, *δ*(ppm)): 12.830 (s, 1H, –N–N**H**–CS–), 12.620 (s, 1H, –N–N**H**–CS–), 7.409–7.250 (m, 10H, aromatic protons), 4.441 (s, 2H, S–C**H_2_**–), 4.436 (s, 2H, S–C**H_2_**–), 2.783 (t, 2H, C**H_2_**–CN–, *J* = 8.4 Hz), 2.495 (t, 2H, C**H_2_**–CN, *J* = 2.0 Hz), 2.163 (s, 3H, –CN–C**H_3_**), 1.349–1.244 (m, 4H, CH_3_C**H_2_**C**H_2_**–), 0.762 (t, 3H, C**H_3_**CH_2_–, *J* = 7.2 Hz); UV-Vis (λ_max_, nm) in DMSO: 270, 340.

*Cu–SBDTC–Heptadione* (**Cu10**)

Yield: 85%; mp: >300 °C; IR (KBr, cm^−1^): 1600 (C=N), 1401, 1135, 1028, 924, 900, 790 (N–N); analytical calculated for [Cu(C_23_H_28_N_4_S_4_)(DMSO)]Cl_2_: %C (42.94), %H (4.61), %N (8.01), %S (22.92); found: %C (42.82), %H (4.38), %N (8.97), %S (23.34); UV-Vis (λ_max_, nm) in DMSO: 268, 334, 509.

#### 3.1.3. Cyclic Voltammetry

Cyclic voltammetry was performed via a BAS CV analyser (Bioanalytical Systems, West Lafayette, IN, USA). Half-wave potentials (*E*_1/2_) were measured for the reduction and oxidation of Cu (II) complexes at a concentration of 28 mM in anhydrous, deoxygenated DMF containing 0.1 M tetrabutylammonium perchlorate as the supporting electrolyte. The scan rate was 100 mVs^−1^. The working electrode was glassy carbon, the counter electrode was Pt wire, and the reference electrode was Ag/AgCl.

#### 3.1.4. X-ray Crystallography 

The crystals for analysis were prepared by the slow evaporation method. Single X-ray crystallography was obtained for the crystals using a SuperNova diffractometer (Agilent Technologies, Santa Clara, CA, USA) with an Atlas CCD detector (Agilent Technologies, Santa Clara, CA, USA). The collected data were reduced using the program SAINT, and an empirical absorption correction was carried out using SADABS. The structure was solved by direct methods using Olex2 [24] and refined by the full-matrix least-squares method using SHELXL [25]. All non-H atoms were anisotropically refined, while hydrogen atoms were positioned geometrically and refined isotropically. The molecular graphics were made using mercury [26]. CCDC 154632 and CCDC 2259882 contained the supplementary crystallographic data for this paper. These data can be obtained free of charge via http://www.ccdc.cam.ac.uk/structures/ (accessed on 25 March 2023) (or from the CCDC, 12 Union Road, Cambridge CB2 1EZ, UK; Fax: +44-1223-336033; E-mail: deposit@ccdc.cam.ac.uk).

### 3.2. Biological Studies

#### 3.2.1. Cell Culture 

MCF-7 and MDA-MB-231 cells were purchased from the American Type Culture Collection (ATCC) (Manassas, VA, USA). Cells were maintained using RPMI 1640 (Sigma-Aldrich, St. Louis, MO, USA) supplemented with 10% foetal bovine serum (FBS) (Gibco, Grand Island, NY, USA). Cells were cultured under humidified atmosphere containing 5% CO_2_ in air at 37 °C.

#### 3.2.2. Cell Viability Assay 

An MTT assay was used to assess the compounds’ cytotoxic activity against MCF-7 and MDA-MB-231 cells. Cells were seeded in 96-well plates at a density of 8000 cells/well and incubated for 24 h at 37 °C to allow the cells to attach. Cells were then treated with the compounds and incubated for 72 h followed by the addition of 5 mg/mL MTT solution (Sigma-Aldrich, St. Louis, MO, USA). The insoluble formazan crystals formed were solubilized in dimethyl sulfoxide (DMSO), and absorbance readings were finally taken at 570 nm via a microplate reader (µ Quant Biotek Instruments, Winooski, VT, USA). 

#### 3.2.3. Target Microbes Used for Antibacterial Assays

*Staphylococcus aureus* (*S. aureus*) (ATCC: 11632), *Kocuria rhizophila* (*K. rhizophila*) (ATCC: 9341), *Bacillus cereus* (*B. cereus*) (ATCC: 10876), *Citrobacter freundii* (*C. freundii*) (ATCC: 8090), *Pseudomonas aeruginosa* (*P. aeruginosa*) (ATCC: 10145) and *Escherichia coli* (*E. coli*) (ATCC: 8739).

#### 3.2.4. Disc Diffusion Qualitative Antibacterial Assay

Bacteria were cultured in Mueller Hinton Broth (MHB) and then later spread over Mueller Hinton Agar (MHA). Sterile paper discs impregnated with 10 µL of the test compounds at 100 mg/mL were then placed on the cultured bacteria followed by incubation for 24 h, except for *P. aeruginosa*, which had an incubation period of 48 h. The resulting zone of inhibition was finally measured for all test compounds. DMSO was used as a negative control.

#### 3.2.5. Minimum Inhibitory Concentration (MIC) and Minimum Bactericidal Concentration (MBC) 

The MIC for the test compounds was determined as previously reported [27]. Briefly, bacteria were cultured in Mueller Hinton Broth (MHB) and then diluted in fresh MHB at a ratio of 1:100. The test samples were then dissolved in 10% DMSO to form a concentration of 100 mg/mL. The compounds were serially diluted to produce final concentrations ranging from 100 mg/mL to 24.4 µg/mL and were then added to the cultured bacteria in a 96-well plate followed by incubation for 24 h. The turbidity of each well was then checked visually, and the lowest compound concentration that resulted in a clear well was regarded as the compound’s minimum inhibitory concentration for that specific bacterial strain.

The MBC was determined as previously reported [28]. Briefly, 10 µL of broth was taken from a series of treated wells containing the cultured bacteria and the test compound at concentrations ≥MIC and placed on nutrient agar. After incubation, the agar plates were visually inspected, and the lowest compound concentration that resulted in the absence of bacterial colony growth was regarded as the MBC.

## 4. Conclusions

In summary, the present study involved synthesising a series of 20 novel macroacyclic Schiff base ligands and Cu (II) complexes. The synthetic compounds were characterised via several analytical techniques, while compounds **4** and **Cu7** were further characterised using X-ray crystallography. The compounds were assessed for their potential bioactivities, and it was found that **Cu1** was the most promising anticancer compound, while **3** demonstrated the highest antibacterial activity. There seems to be no clear trend or pattern between the analogues’ bioactivity and their chain length, but in general it may be deduced that SMDTC-derived analogues possess higher bioactivity than their SBDTC-derived counterparts. Furthermore, the Cu (II) complexes seemed to possess limited bioactivity, specifically against bacteria, due to their low solubility. This is the first comprehensive study that investigated the biological effects of macroacyclic Schiff bases and their Cu (II) complexes derived from SMDTC and SBDTC and provided a comparison between chain length, SMDTC and SBDTC whenever possible. Moreover, highly active molecules that possessed bioactivities similar to or even more potent than established drugs such as cisplatin and chloramphenicol were identified in this study. These results show the potential of dithiocarbazate-derived macroacyclic Schiff base ligands and their Cu (II) complexes as highly active molecules that may be developed further into potent anticancer and antibacterial drugs.

## Figures and Tables

**Table 1:** Crystallographic data and structure refinement details.

Compounds	4	Cu7
**Chemical formula**	C_20_H_22_N_4_S_4_	CuC_10_H_16_ N_4_S_4_
***M_r_***	446.66	384.05
**Crystal colour**	Dark brown	Brown
**Crystal habit**	Rod	Needle
**Crystal size (mm^3^)**	0.50 × 0.25 × 0.16	0.21 × 0.19 × 0.14
**Temperature (K)**	293	100
**λ (** **Å** **)**	0.71073	1.54178
**Crystal system**	monoclinic	orthorhombic
**Space group**	*P* 2_1_/*n*	*Pbca*
***a* (Å)**	8.2086 (5)	9.2009 (4)
***b* (Å)**	5.4551 (3)	9.5250 (4)
***c* (Å)**	24.6907 (12)	35.8100 (13)
**α (°)**	90.000	90.000
**β (°)**	95.035 (5)	90.000
**γ (°)**	90.000	90.000
***V* (Å^3^)**	1101.34 (10)	3138.3 (2)
***Z***	1	8
**Temperature (K)**	293 (2)	100 (2)
**F(000)**	468	1576
**μ (mm^−1^)**	0.445	6.870
***θ*_min_ (°)**	3.1042	4.940
***θ*_max_ (°)**	29.2305	71.371
**No. of measured, independent and observed [I > 2σ(I)] reflections**	11384, 2049, 1759	6741, 2965, 2460
***R*_int_**	0.127	0.031
**Goodness of fit (GOF) on F^2^**	1.09	1.061
***R*[*F*^2^ > 2σ(*F*^2^)], *wR*(*F*^2^), *S***	0.084, 0.227, 1.09	0.036, 0.093, 1.06
**No. of reflections**	2049	2965
**No. of parameters**	128	173
**Δρ_max_ (e Å^−3^)**	2.20	0.34
**Δρ_min_ (e Å^−3^)**	−1.50	−0.27

**Table 2:** Selected geometric parameters of **4** and **Cu7** (Å, °).

**Compound 4**			
C3–S1	1.659 (5)	C3–N2	1.352 (7)
C3–S2	1.735 (5)	N1–N2	1.364 (6)
C2–N1	1.294 (9)	C1–C2	1.517 (9)
C3–S2–C4	100.3 (2)	S2–C4–C5	110.3 (3)
C3–N2–N1	118.2 (4)	N2–C3–S1	121.4 (4)
**Compound Cu7**			
Cu1–S3	2.2432 (9)	Cu1–N3	1.963 (3)
N1–N2	1.382 (4)	C5–N3	1.293 (4)
C2–N1	1.309 (4)	C9–S3	1.752 (3)
N3–Cu1–S2	164.42 (9)	C9–N4–N3	111.1 (3)
N2–Cu1–S3	164.99 (9)	S4–C9–S3	113.4 (2)

**Table 3:** Hydrogen bond interactions of compound **4** (Å, °).

*Cg* is the Centroid of the Ring (C5–C6–C7–C8–C9–C10)				
*D*–H···*A*	*D*–H	H···*A*	*D*···*A*	*D*–H···*A*
N2–H2···S1 ^i^	0.86	2.63	3.480 (5)	171
C9–H9···*Cg* ^ii^	0.93	2.74	3.466 (5)	136

Symmetry codes: (i) −*x* + 1, −*y* + 1, −*z*; (ii) −*x* + 5/2, *y*−1/2, −*z* + 1/2.

**Table 4:** Hydrogen bond interactions of compound **Cu7** (Å, °).

*Cg* is the Centroid of the Ring (C5–C6–C7–C8–C9–C10)				
*D*–H···*A*	*D*–H	H···*A*	*D*···*A*	*D*–H···*A*
C1–H1C···S1 ^i^	0.98	2.86	3.652 (4)	138

Symmetry codes: (i) *x* + 1/2, *y*, −*z* + 1/2.

**Table 5:** Electrochemical data for the Cu (II) complexes vs. AgCl/Ag.

Compound	*E*_pc_ (V)	*E*_pa_ (V)	Δ*E*_p_ (mV)
**Cu1**	−0.072	0.107	179
**Cu2**	−0.043	0.120	163
**Cu3**	−0.250	−0.115	135
**Cu4**	−0.231	−0.096	135
**Cu5**	−0.261	−0.065	196
**Cu6**	−0.205	−0.091	114
**Cu7**	−0.250	−0.078	172
**Cu8**	−0.212	−0.078	134
**Cu9**	−0.249	−0.070	179
**Cu10**	−0.210	−0.064	146

**Table 6:** IC_50_ values of the Schiff base ligands and their macroacyclic Cu (II) complexes.

Compound	IC_50_ (µM) ^a^	
	MCF-7	MDA-MB-231
**1**	6.9 ± 0.3	>50
**Cu1**	1.7 ± 0.1	1.4 ± 0.1
**2**	2.6 ± 0.8	4.5 ± 0.7
**Cu2**	>50	>50
**3**	20 ± 0.3	4.2 ± 0.7
**Cu3**	46 ± 1.0	19 ± 4.1
**4**	49 ± 5.4	>50
**Cu4**	11 ± 1.9	38 ± 7.5
**5**	17 ± 1.5	22 ± 2.0
**Cu5**	14 ± 2.1	16 ± 4.6
**6**	22 ± 1.2	>50
**Cu6**	>50	9 ± 1.2
**7**	12 ± 0.4	4 ± 0.2
**Cu7**	45 ± 2.3	>50
**8**	7.3 ± 0.8	12 ± 1.1
**Cu8**	7.3 ± 2.8	12 ± 0.5
**9**	9.8 ± 0.1	6 ± 0.2
**Cu9**	20 ± 1.5	>50
**10**	5.7 ± 0.1	22 ± 0.8
**Cu10**	>50	>50
**Cisplatin**	25 ± 0.3	48 ± 3.5

^a^ IC_50_ values are reported as the mean (IC_50_ ± SD) of duplicates of three independent experiments.

**Table 7:** IC_50_ values of the Schiff base ligands and their macroacyclic Cu (II) complexes.

Compound	Zone of Inhibition Diameter (mm) ^a^					
	*S. aureus*	*B. cereus*	*K. rhizophila*	*E. coli*	*P. aeruginosa*	*C. freundii*
**1**	0	0	0	0	0	0
**Cu1**	0	0	0	0	0	0
**2**	0	7.5	8.5	8	0	0
**Cu2**	0	8	8	0	0	0
**3**	0	0	25	0	0	0
**Cu3**	12	9	9	0	0	0
**4**	13	11	15	0	0	0
**Cu4**	0	10	10	0	0	0
**5**	17	11	22	0	0	0
**Cu5**	9	0	8	0	0	0
**6**	0	0	0	0	0	0
**Cu6**	0	0	0	0	0	0
**7**	0	0	0	0	0	0
**Cu7**	0	0	15	0	7	0
**8**	0	0	0	0	0	0
**Cu8**	0	0	0	0	0	0
**9**	0	0	0	0	0	0
**Cu9**	0	0	15	0	8	0
**10**	10.5	0	0	0	0	0
**Cu10**	0	0	0	0	0	0
**Chloramphenicol**	25	18.7	19.3	24.3	27.3	21

^a^ Zone of inhibition diameters equal to 15 mm and above were considered to be significant.

**Table 8:** MIC and MBC of the most active Schiff bases and Cu (II) complexes against *S. aureus* and *K. rhizophila*.

Compound	MIC (µg/mL)		MBC (µg/mL)	
	*S. aureus*	*K. rhizophila*	*S. aureus*	*K. rhizophila*
**3**	<24.4	<24.4	<24.4	48.8
**4**	-	1562.5	-	3125
**5**	-	781.25	-	1562.5
**Cu7**	-	<24.4	-	48.8
**Cu9**	-	<24.4	-	48.8
**Chloramphenicol**	<24.4	<24.4	-	-

(-) refers to absence of measurable inhibitory action at 100 mg/mL.

## Data Availability

The data presented in this study are available upon request from the corresponding author.

## Supplementary Materials

The following supporting information can be downloaded at: https://www.mdpi.com/article/10.3390/molecules28135009/s1.

## Sample Availability

Samples of the compounds are available from the authors.
