# Supplementary material for: Synthesis, Crystal Structure, Antibacterial and In Vitro Anticancer Activity of Novel Macroacyclic Schiff Bases and Their Cu (II) Complexes Derived from S-Methyl and S-Benzyl Dithiocarbazate
Source: Molecules. 2023 Jun 26;28(13):5009. doi: 10.3390/molecules28135009 (PMC10343930; doi:10.3390/molecules28135009)
Supplement: Supplementary file 1 [file molecules-28-05009-s001.zip › molecules-2436624-supplementary.pdf]

NMR Spectra

SMDTC-glyoxal ***(1)***

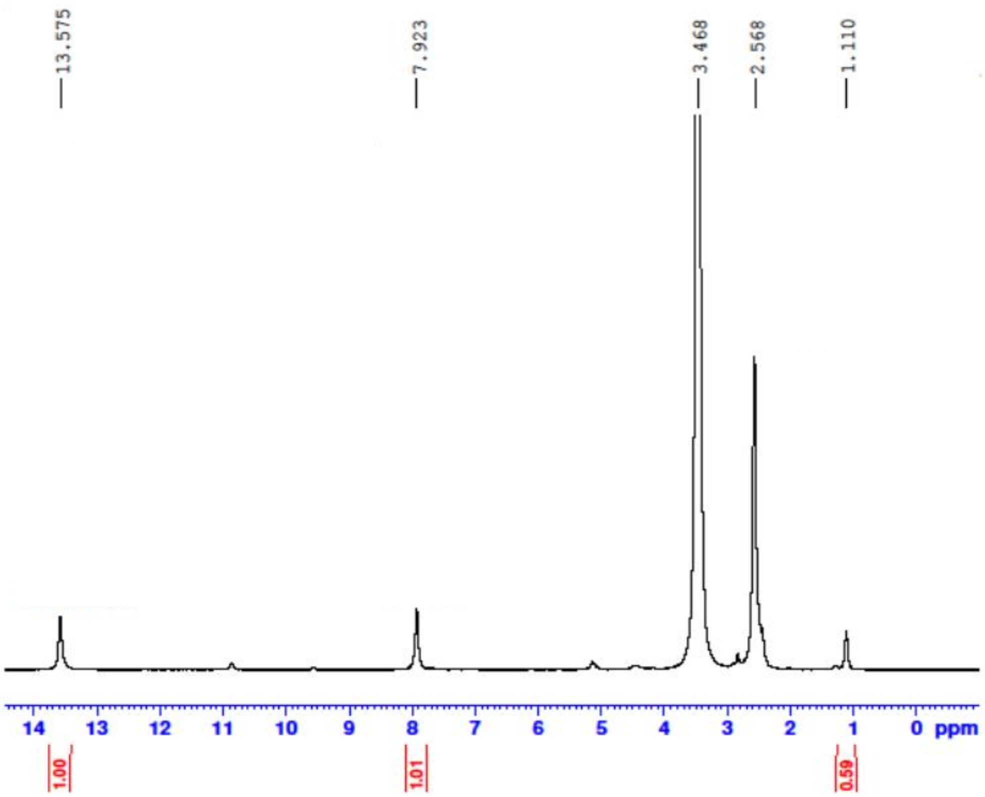

Figure S1. NMR Spectrum of compound **1**

SBDTC-glyoxal **(2)**

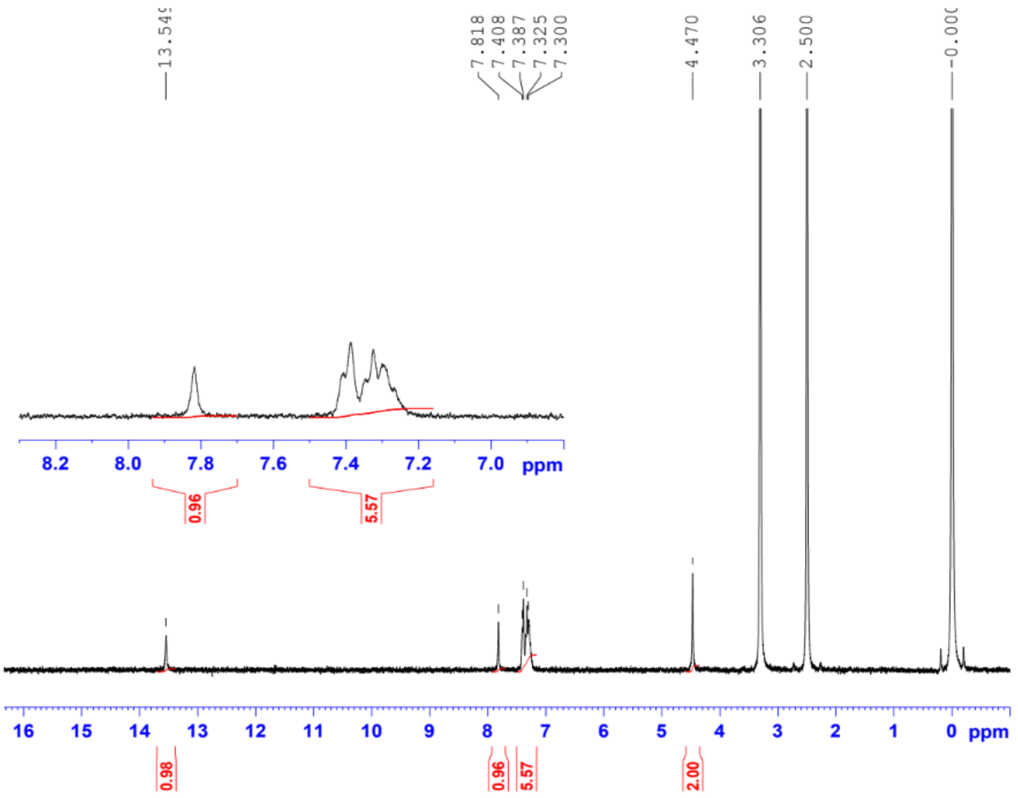

Figure S2. NMR Spectrum of compound **2**

SMDTC-Butanedione **(3)**

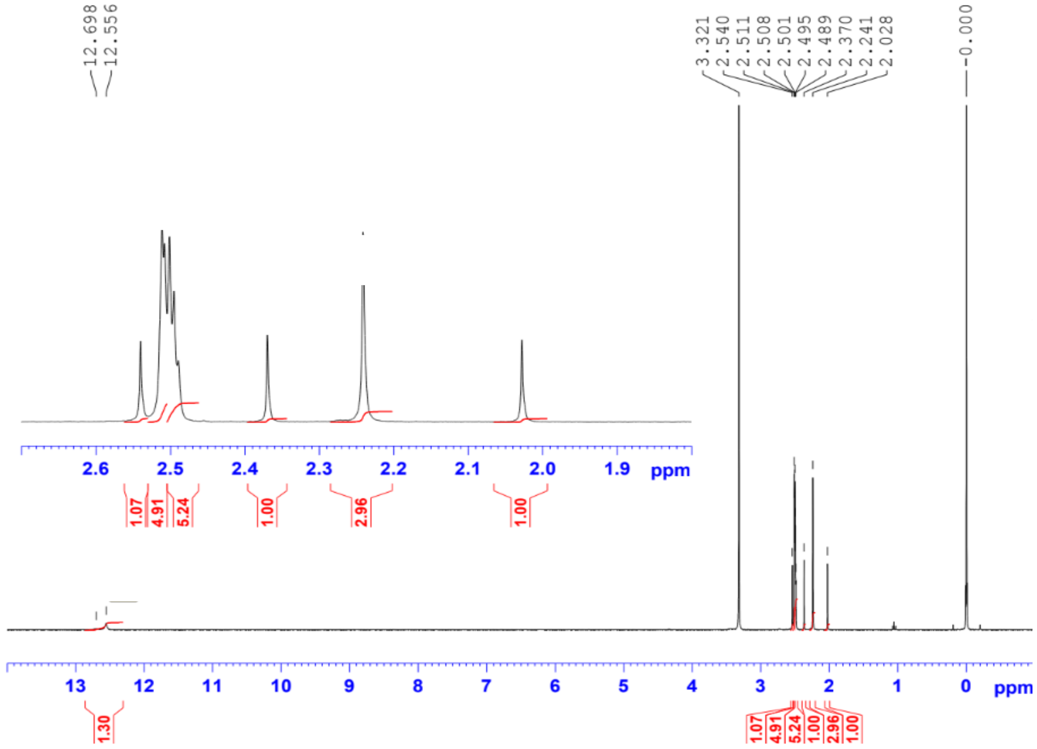

Figure S3. NMR Spectrum of compound **3**

SBDTC-Butanedione ***(4)***

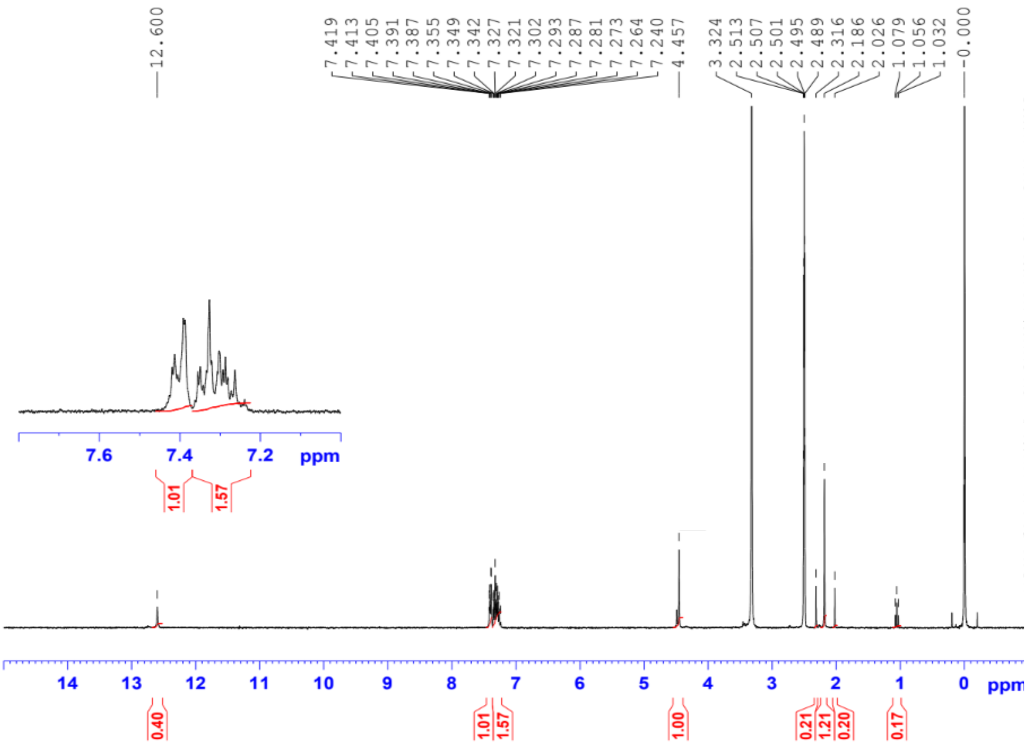

Figure S4. NMR Spectrum of compound **4**

SMDTC-Pentadione **(5)**

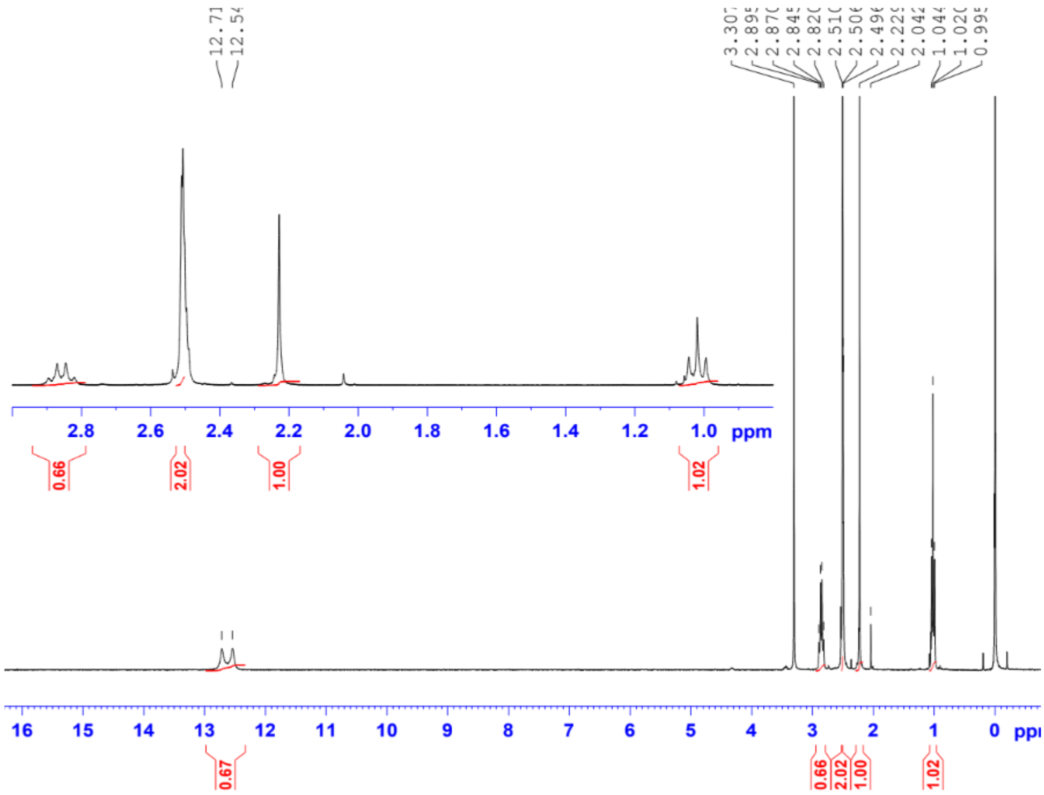

Figure S5. NMR Spectrum of compound **5**

SBDTC-Pentadione ***(6)***

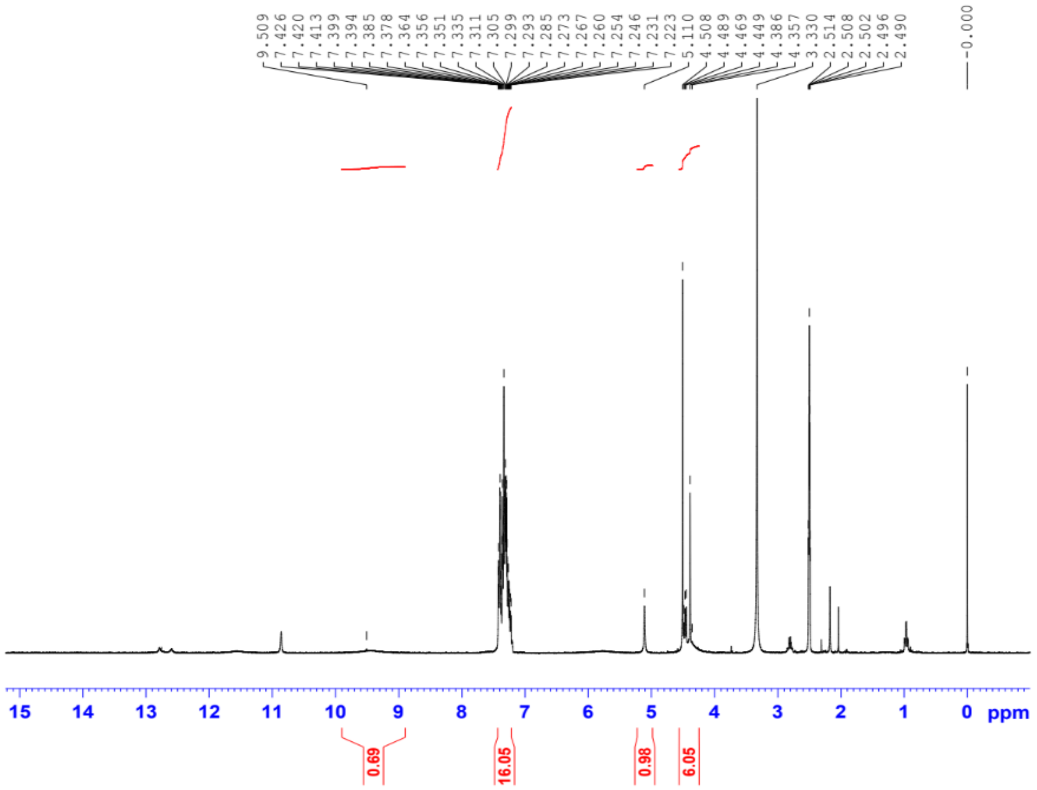

Figure S6. NMR Spectrum of compound **6**

SMDTC-Hexadione ***(7)***

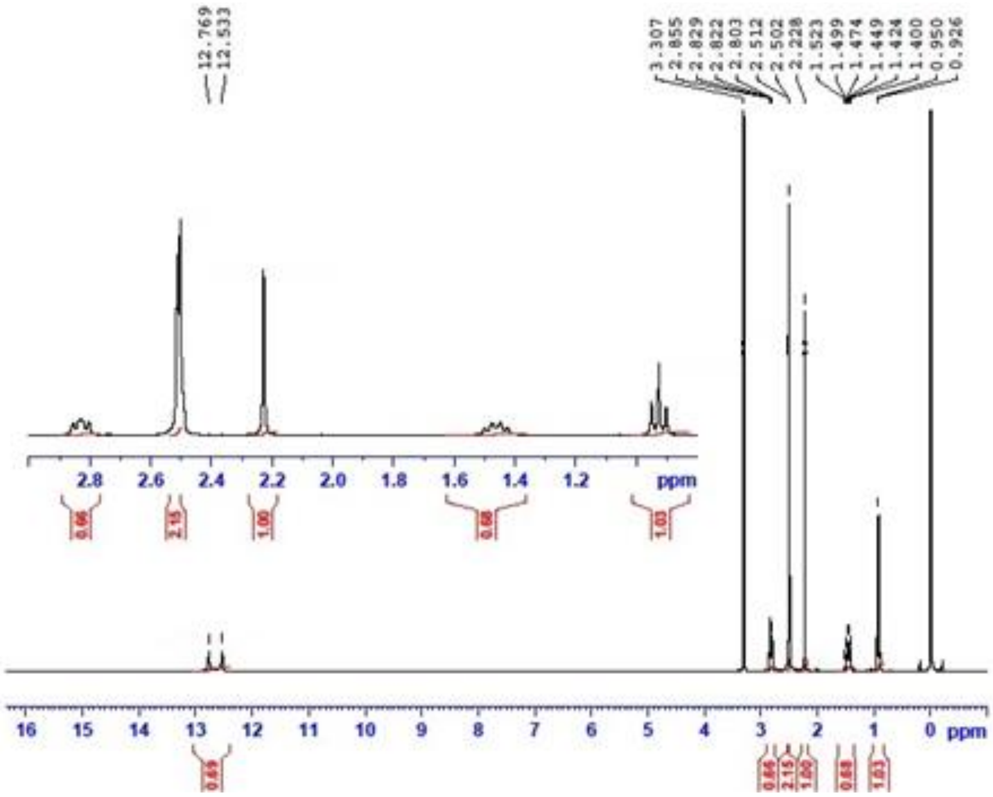

Figure S7. NMR Spectrum of compound **7**

SBDTC-Hexadione ***(8)***

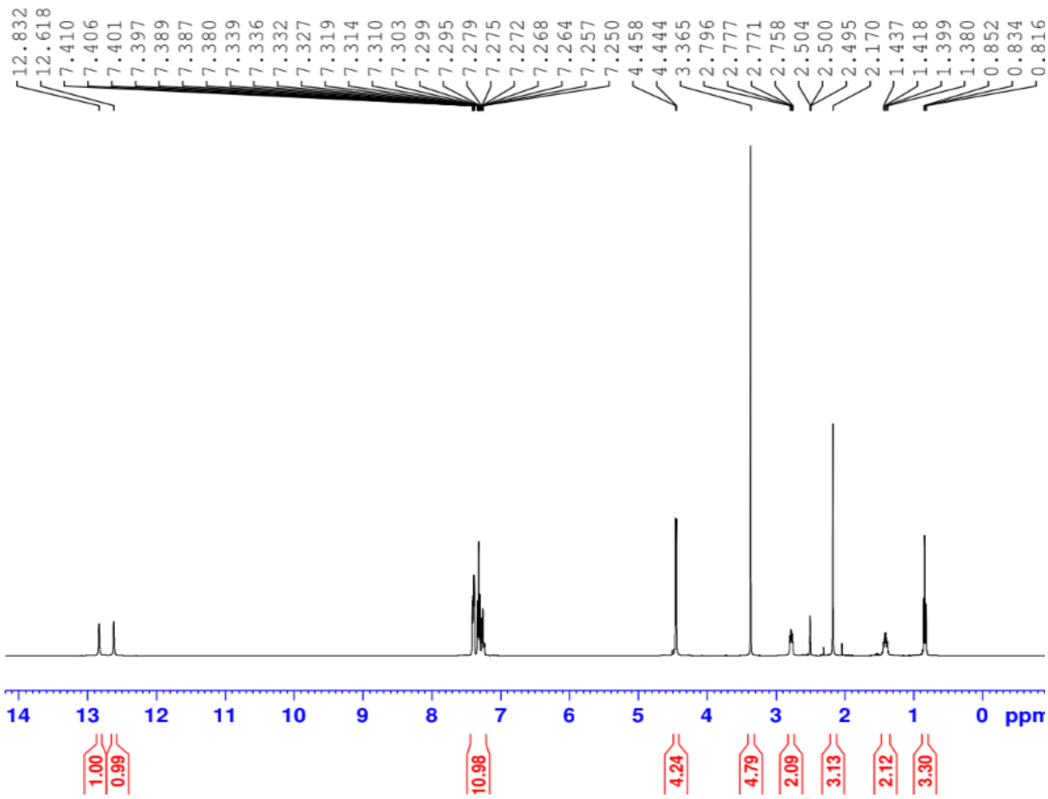

Figure S8. NMR Spectrum of compound **8**

SMDTC-Heptadione ***(9)***

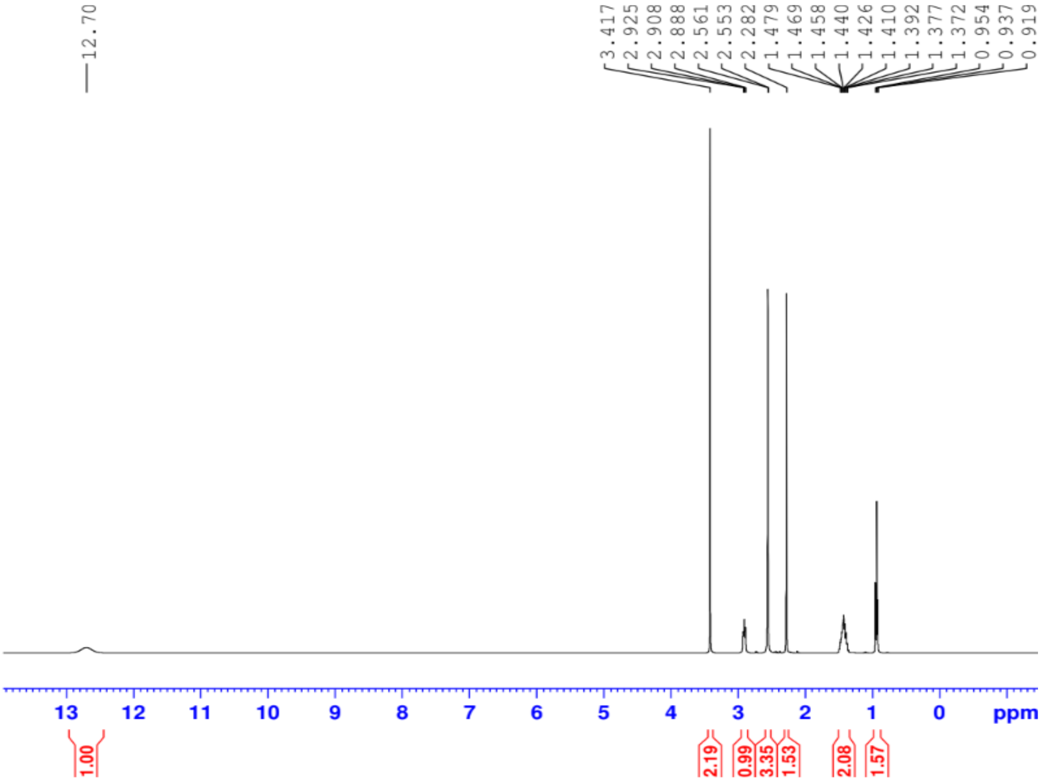

Figure S9. NMR Spectrum of compound **9**

SBDTC-Heptadione ***(10)***

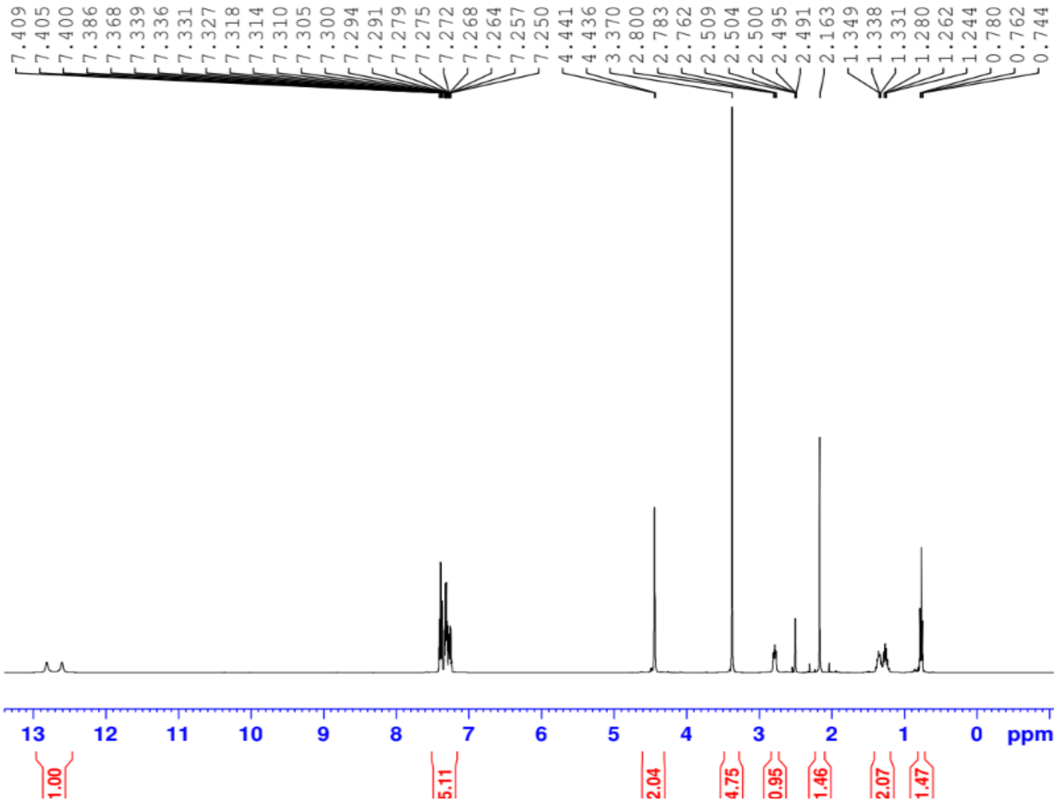

Figure S10. NMR Spectrum of compound **10**

FTIR Spectra

SMDTC-glyoxal ***(1)***

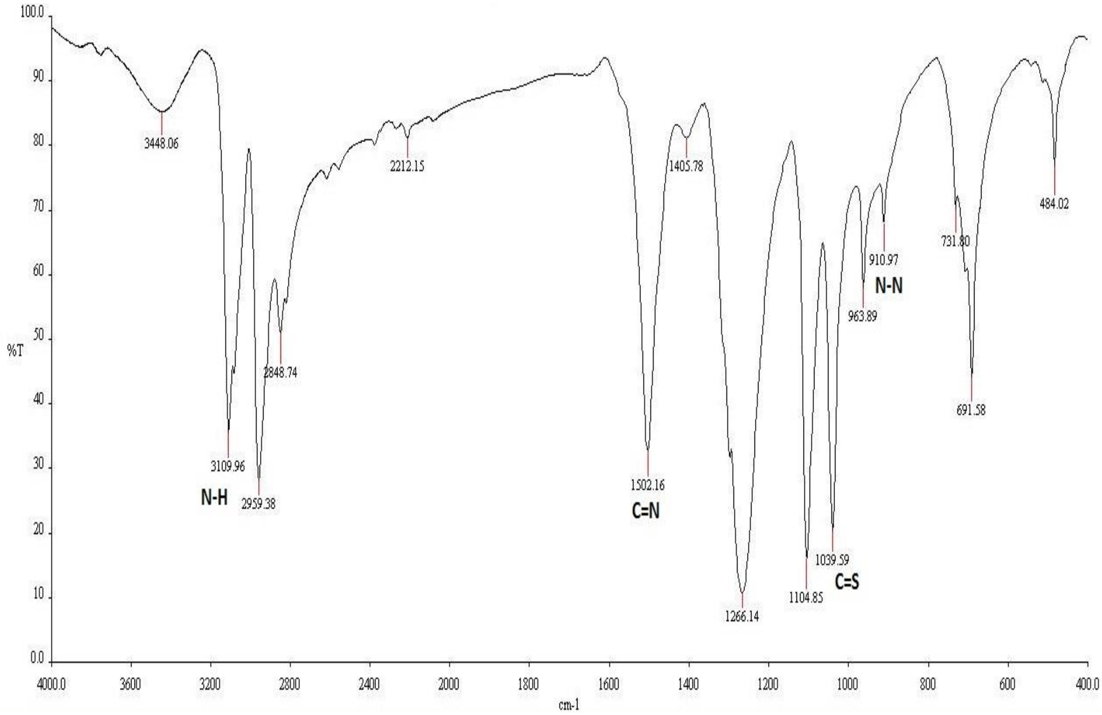

Figure S11. FTIR Spectrum of compound **1**

Cu-SMDTC-glyoxal ***(Cu1)***

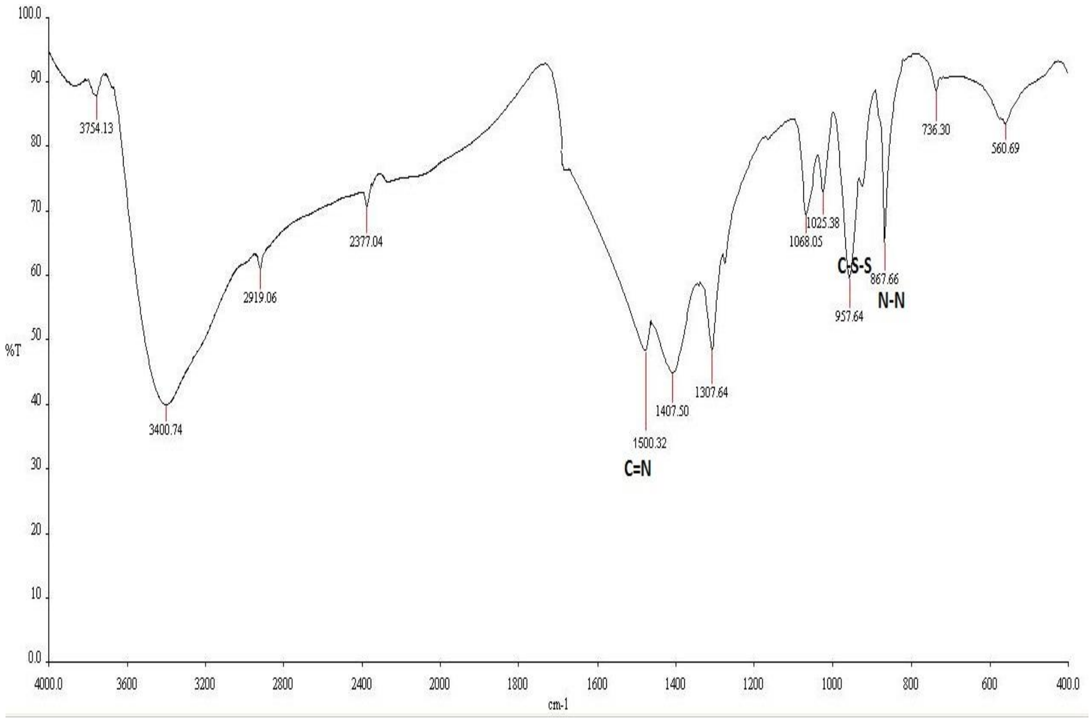

Figure S12. FTIR Spectrum of compound **Cu1**

SBDTC-glyoxal ***(2)***

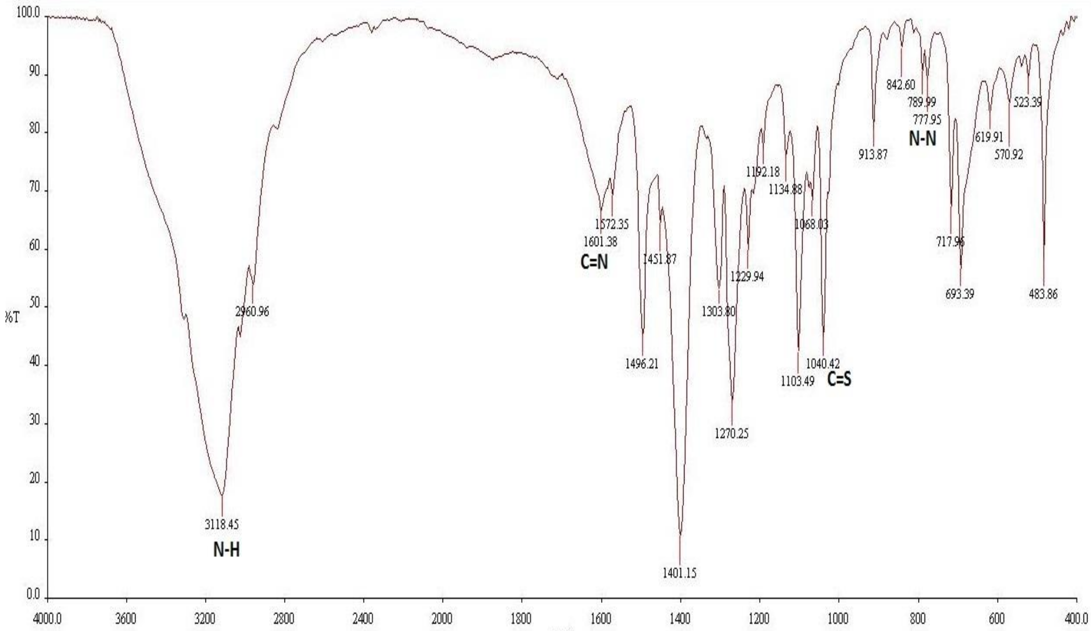

Figure S13. FTIR Spectrum of compound **2**

Cu-SBDTC-glyoxal ***(Cu2)***

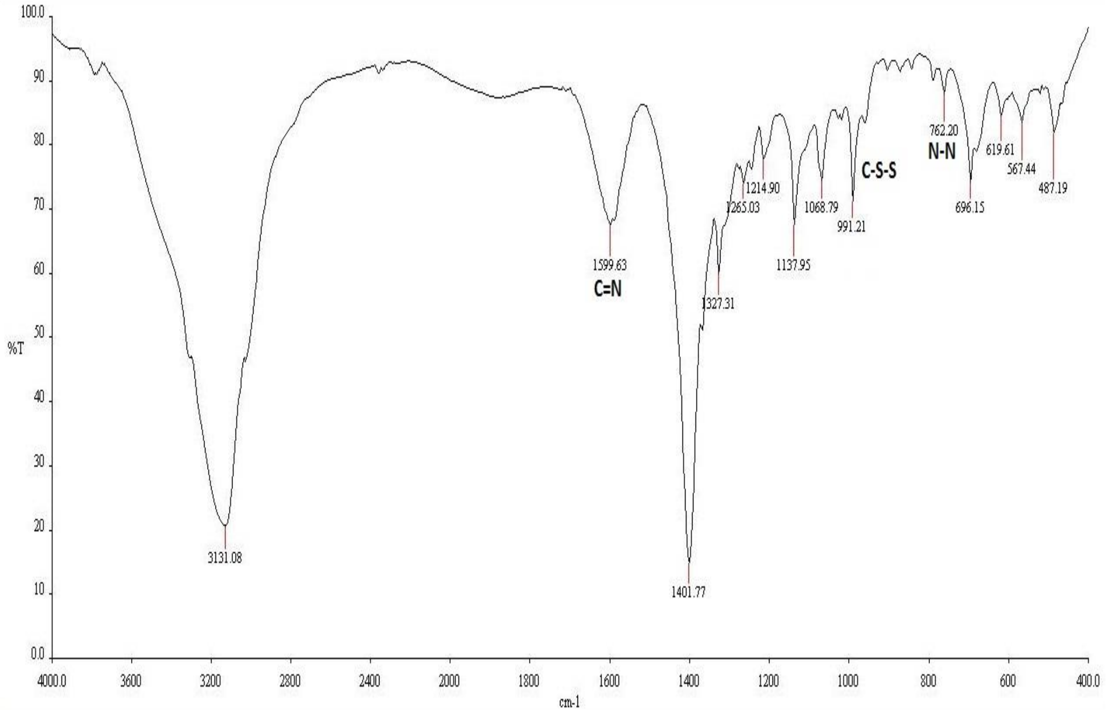

Figure S14. FTIR Spectrum of compound **Cu2**

SMDTC-Butanedione ***(3)***

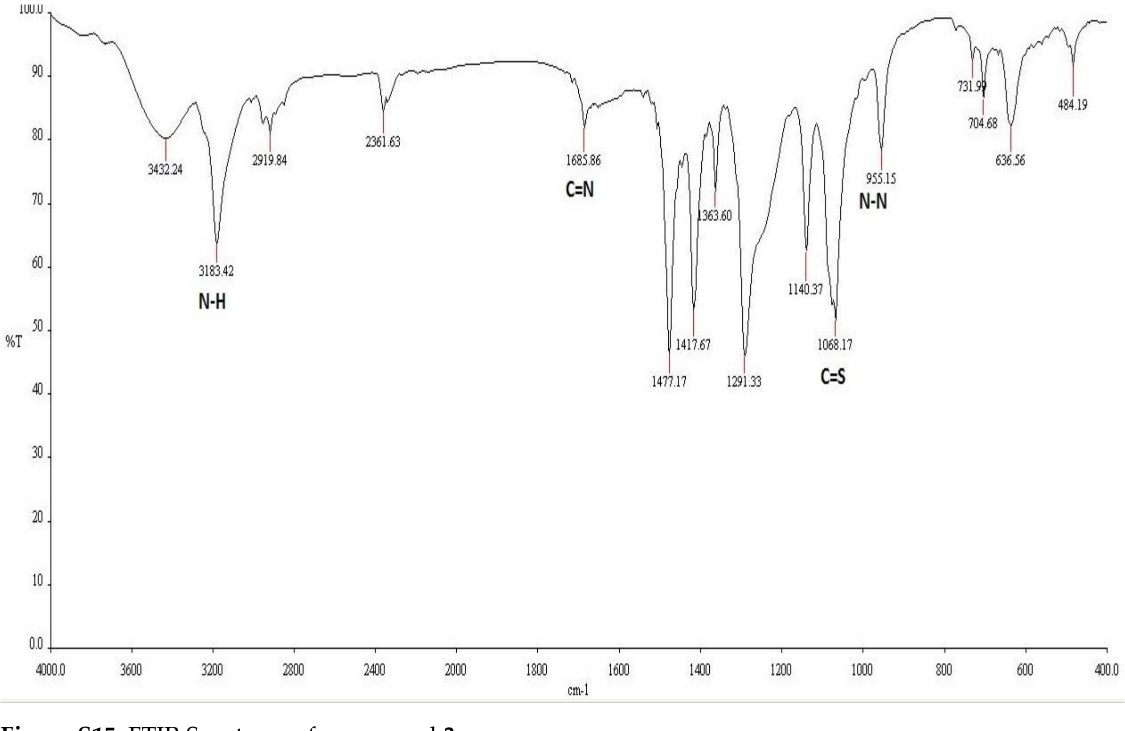

Figure S15. FTIR Spectrum of compound **3**

Cu-SMDTC-Butanedione ***(Cu3)***

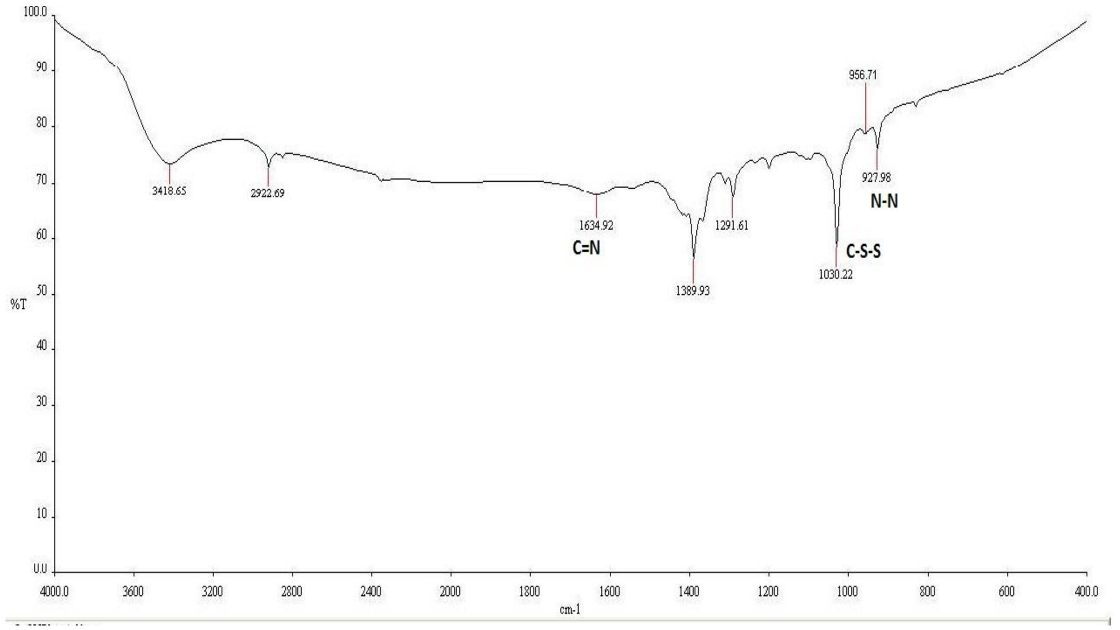

Figure S16. FTIR Spectrum of compound **Cu3**

SBDTC-Butanedione ***(4)***

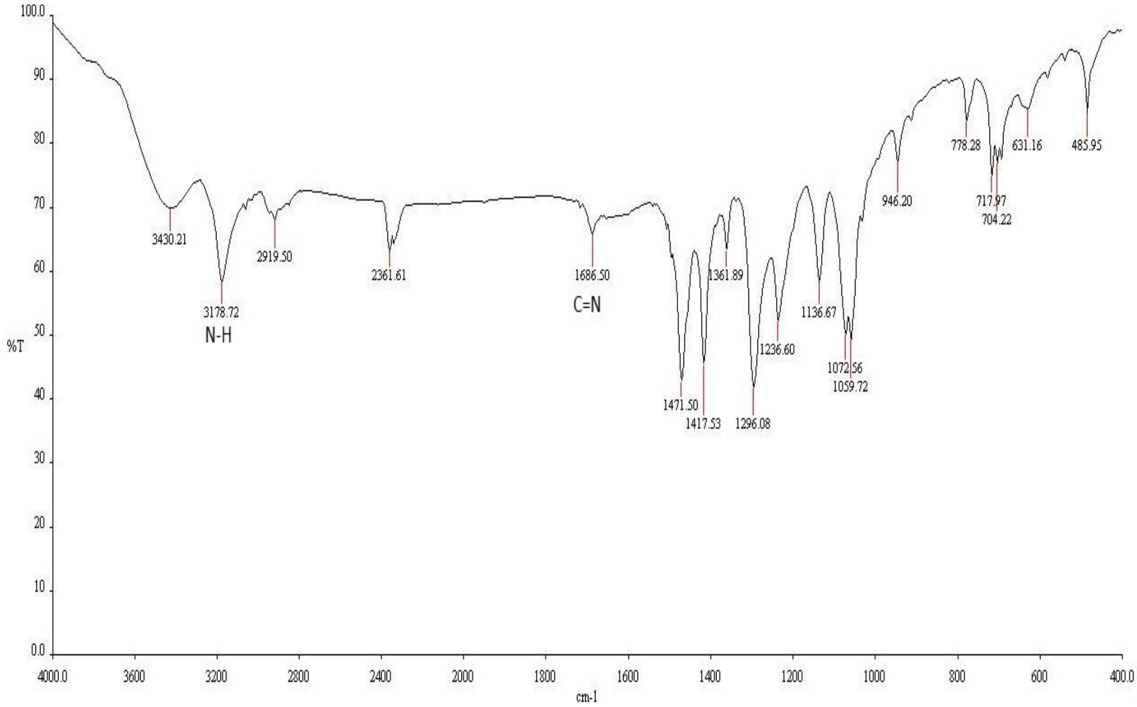

Figure S17. FTIR Spectrum of compound **4**

*Cu-SBDTC-Butanedione **(Cu4)***

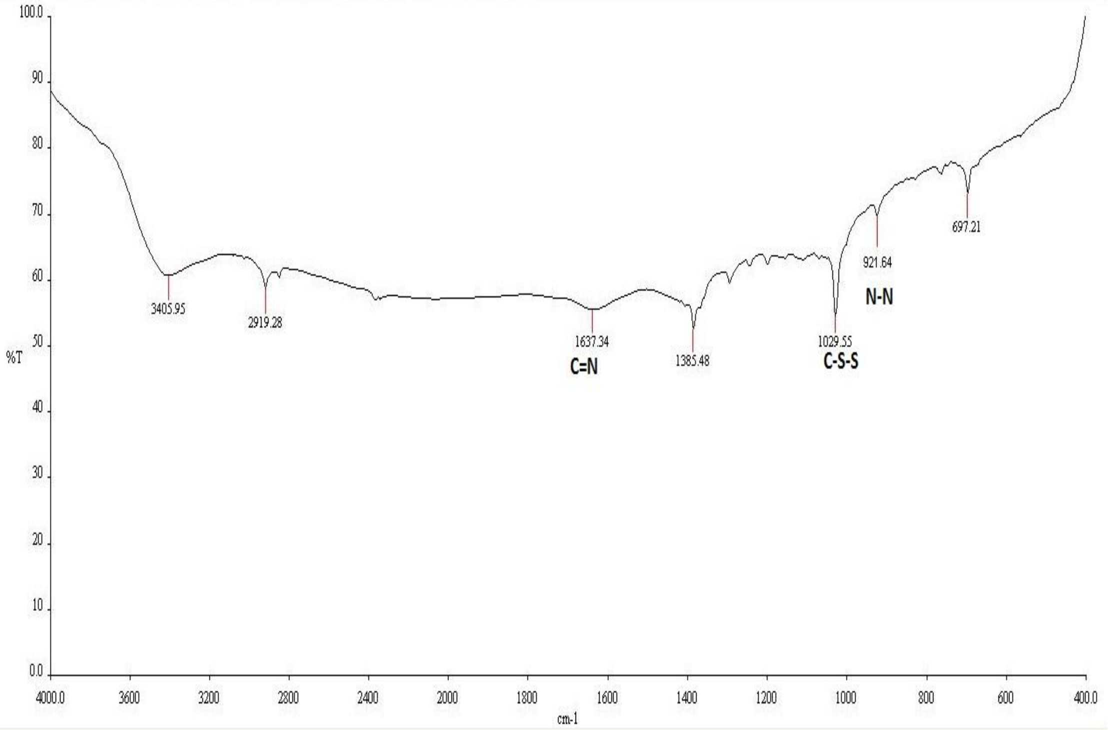

Figure S18. FTIR Spectrum of compound **Cu4**

*SMDTC-Pentadione **(5)***

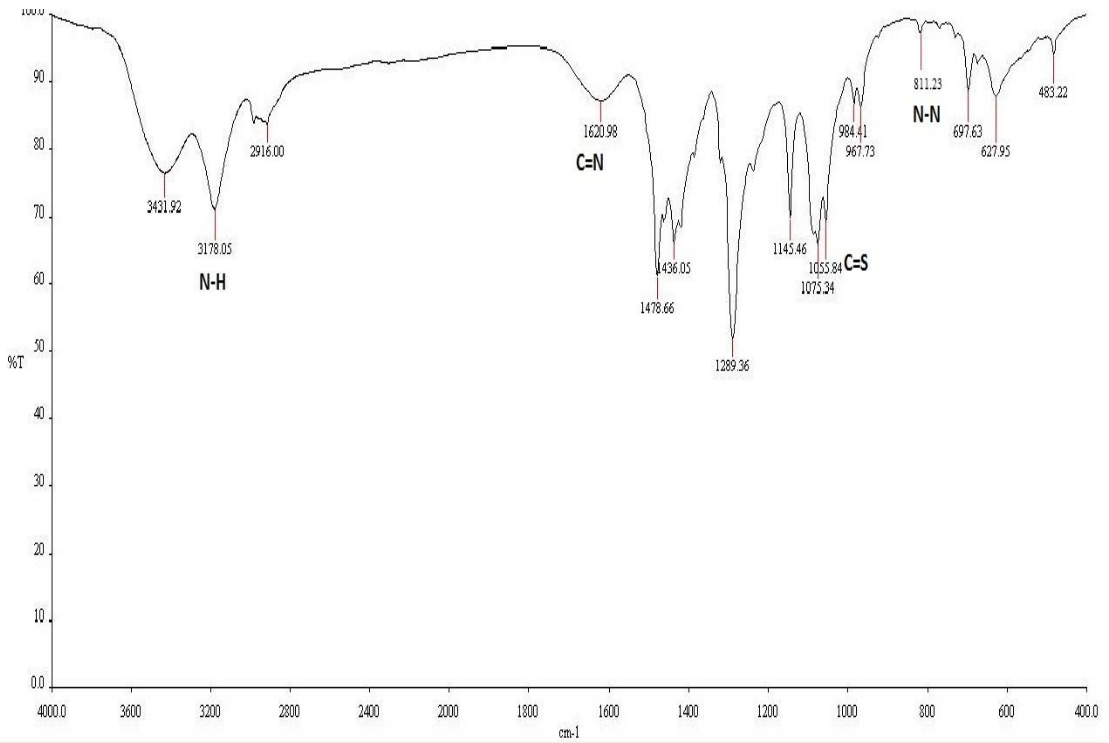

Figure S19. FTIR Spectrum of compound **5**

Cu-SMDTC-Pentadione ***(Cu5)***

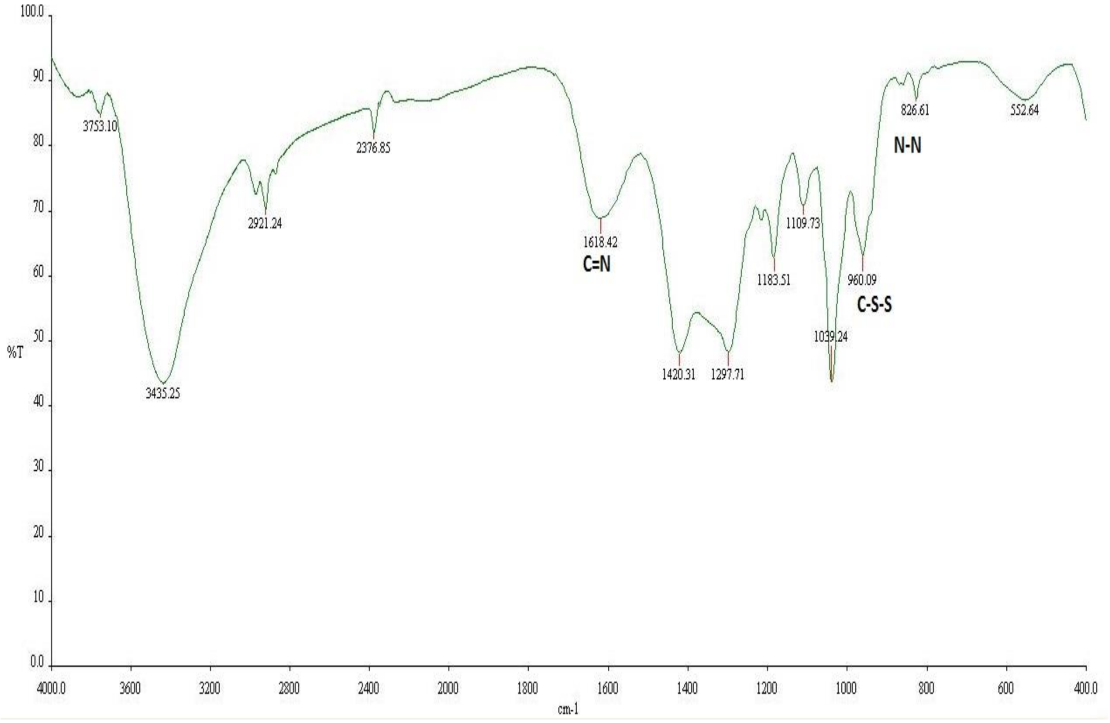

Figure S20. FTIR Spectrum of compound **Cu5**

SBDTC-Pentadione ***(6)***

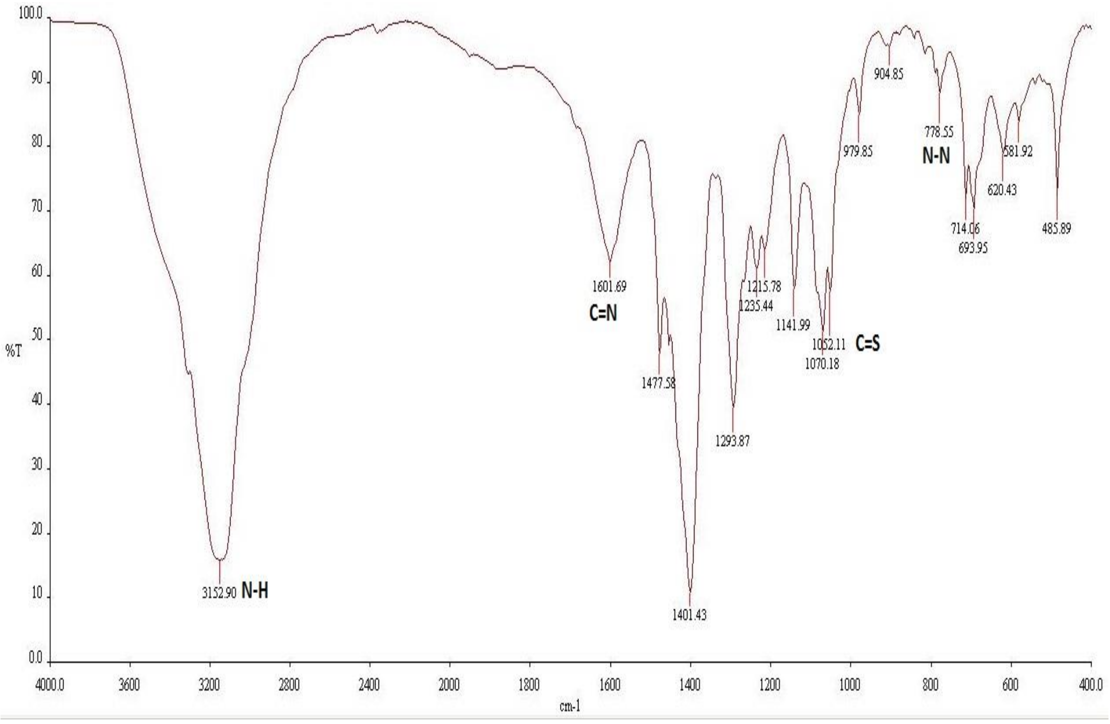

Figure S21. FTIR Spectrum of compound **6**

Cu-SBDTC-Pentadione ***(Cu6)***

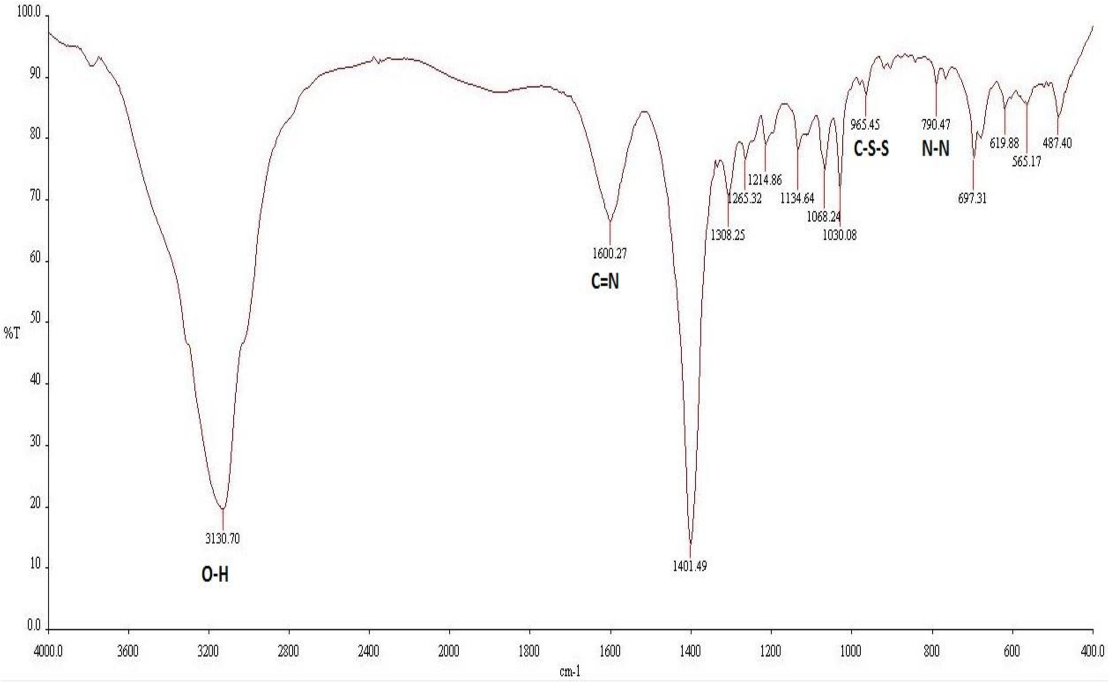

Figure S22. FTIR Spectrum of compound **Cu6**

SMDTC-Hexadione ***(7)***

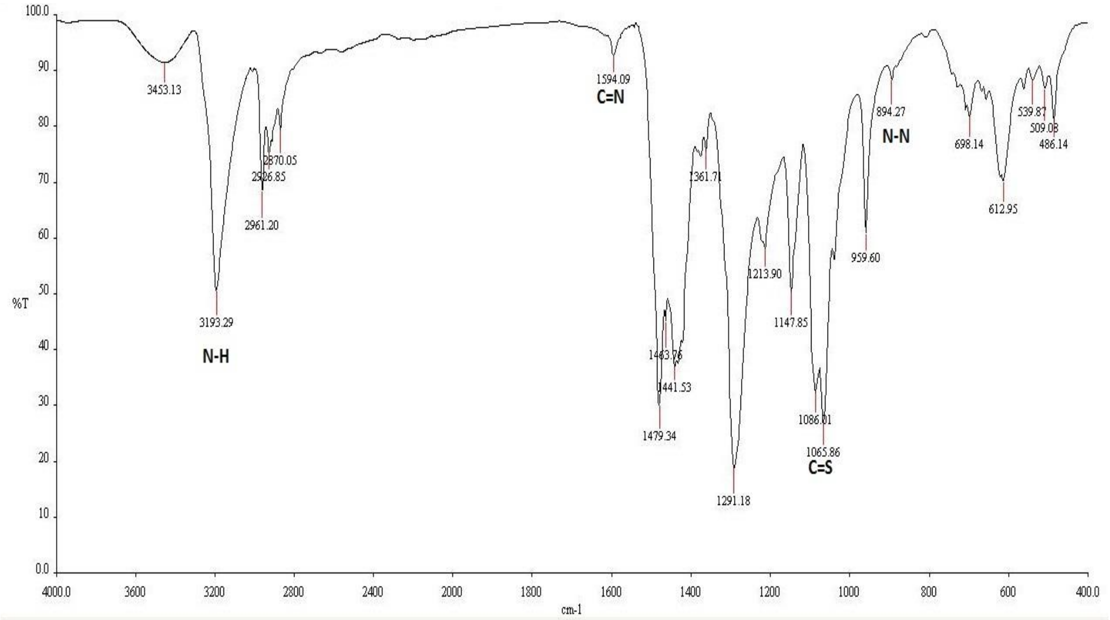

Figure S23. FTIR Spectrum of compound **7**

Cu-SMDTC-Hexadione ***(Cu7)***

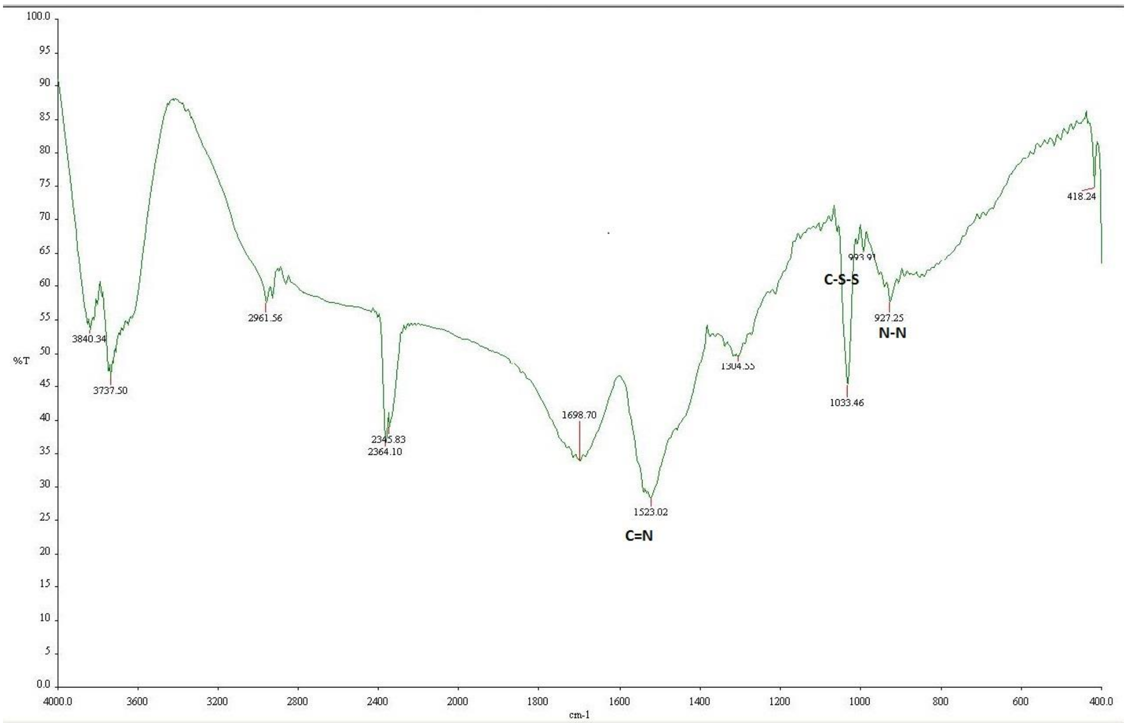

Figure S24. FTIR Spectrum of compound **Cu7**

SBDTC-Hexadione ***(8)***

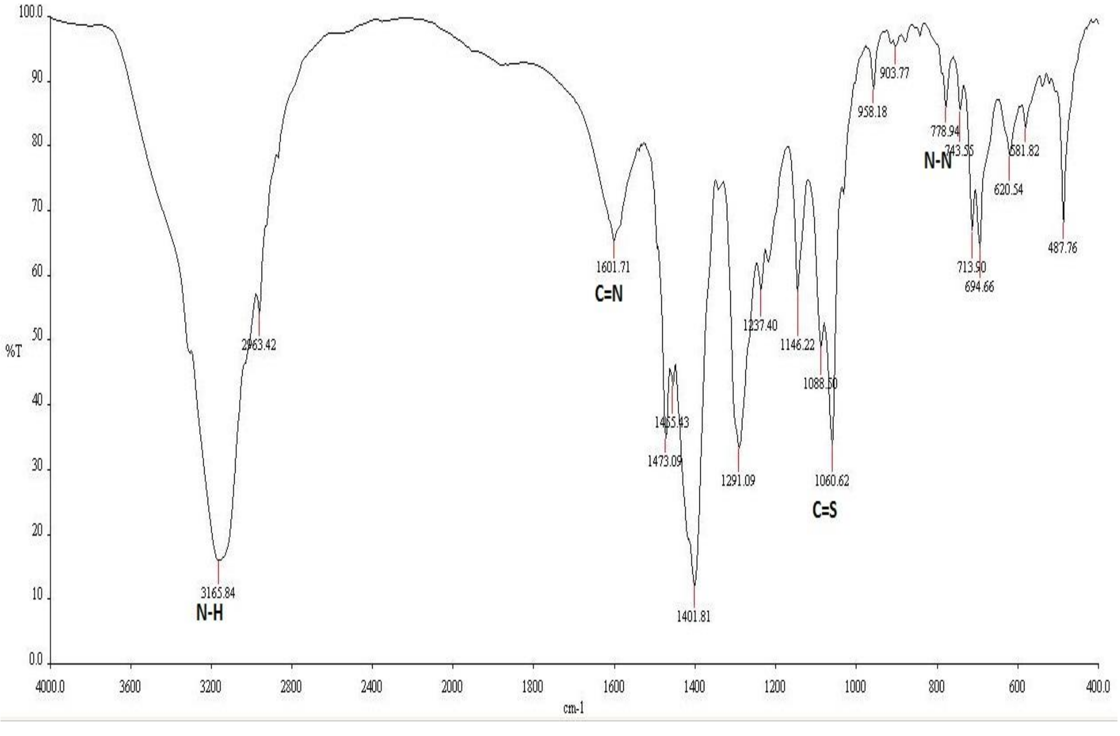

Figure S25. FTIR Spectrum of compound **8**

Cu-SBDTC-Hexadione ***(Cu8)***

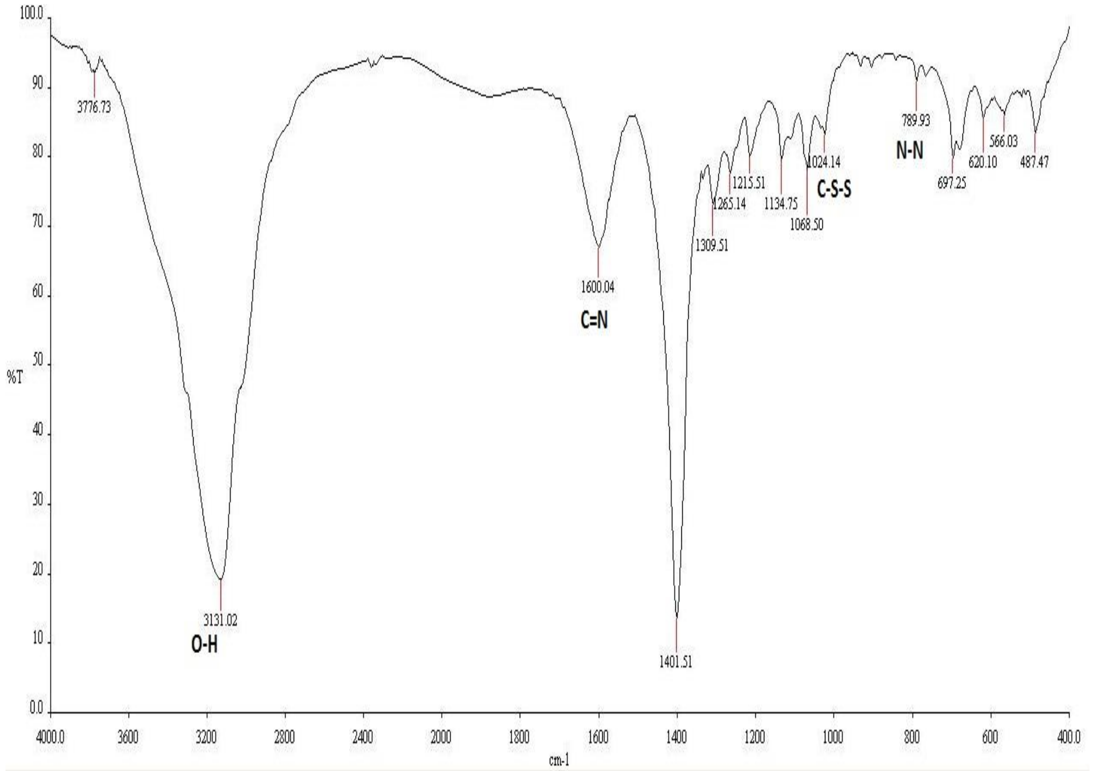

Figure S26. FTIR Spectrum of compound **Cu8**

SMDTC-Heptadione ***(9)***

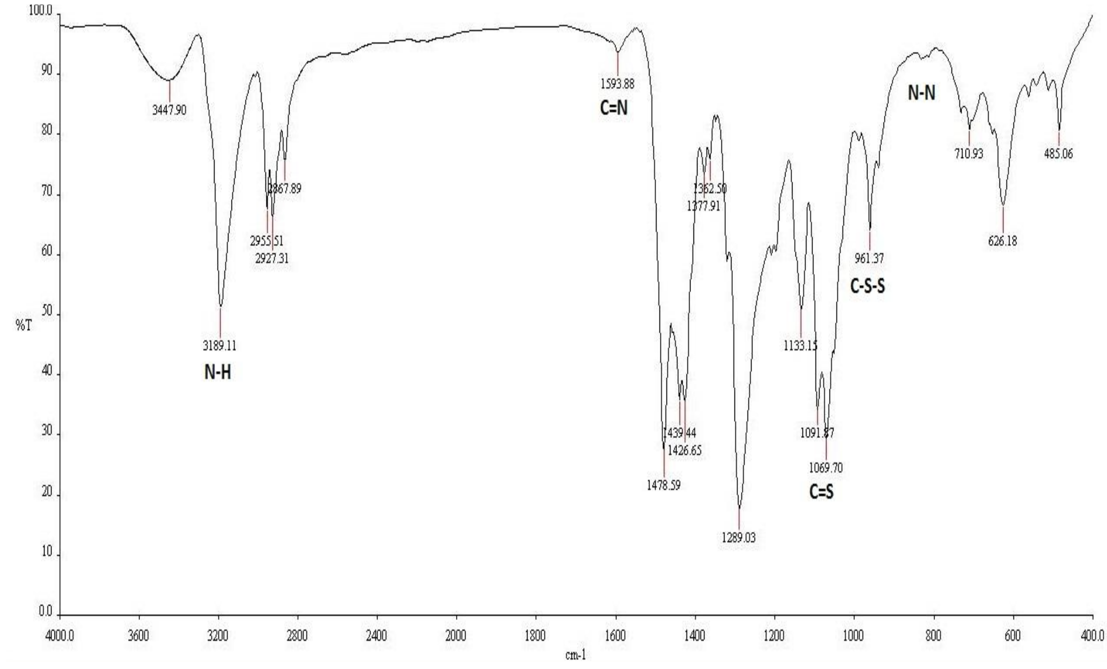

Figure S27. FTIR Spectrum of compound **9**

Cu-SMDTC-Heptadione ***(Cu9)***

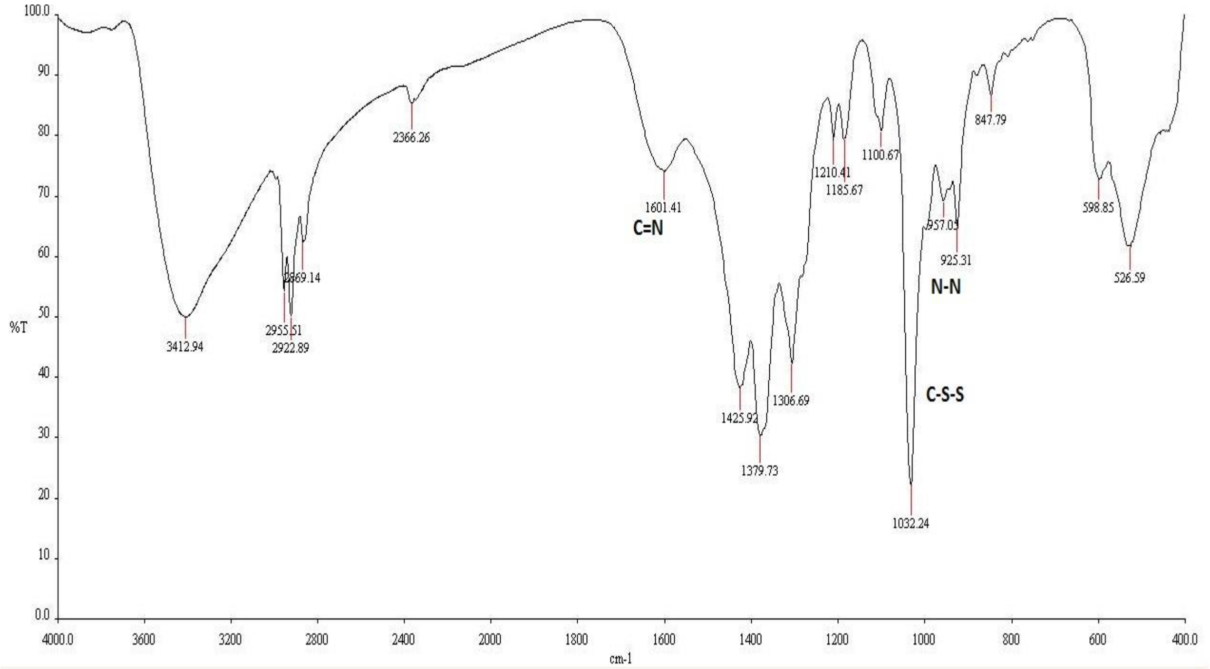

Figure S28. FTIR Spectrum of compound **Cu9**

SBDTC-Heptadione ***(10)***

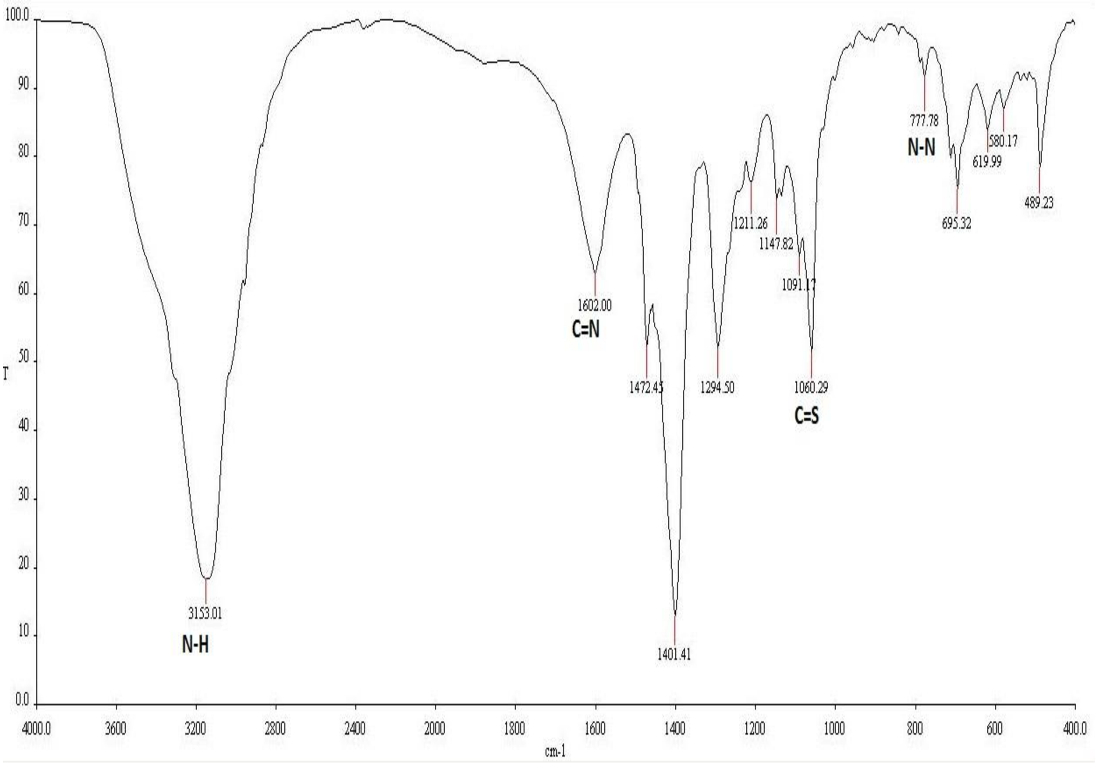

Figure S29. FTIR Spectrum of compound **10**

Cu-SBDTC-Heptadione ***(Cu10)***

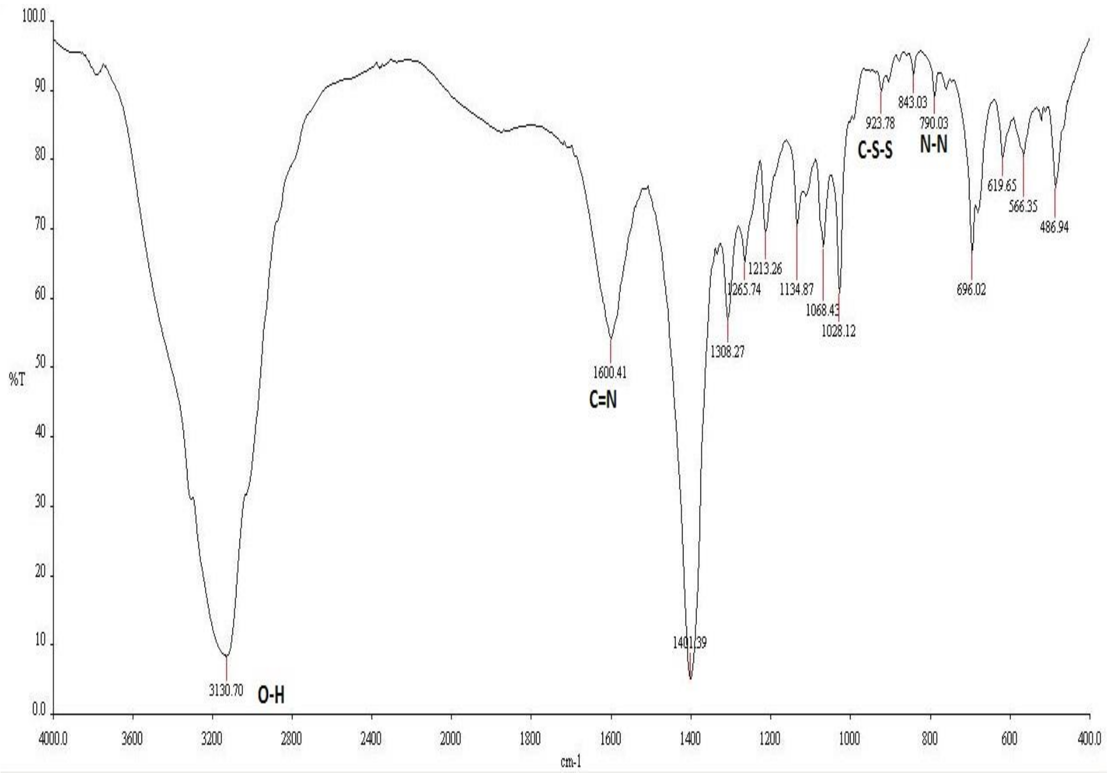

Figure S30. FTIR Spectrum of compound **Cu10**
